# Tunable Emissive CsPbBr_3_/Cs_4_PbBr_6_ Quantum Dots Engineered by Discrete Phase Transformation for Enhanced Photogating in Field‐Effect Phototransistors

**DOI:** 10.1002/advs.202401973

**Published:** 2024-06-21

**Authors:** Xiao Han, Siyuan Wan, Lin He, Junlong Zou, Andraz Mavric, Yixi Wang, Marek Piotrowski, Anil Kumar Bandela, Paolo Samorì, Zhiming Wang, Tim Leydecker, Udayabhaskararao Thumu

**Affiliations:** ^1^ Institute of Fundamental and Frontier Sciences University of Electronic Science and Technology of China Chengdu 610054 China; ^2^ Materials Research Laboratory University of Nova Gorica Vipavska 13 Nova Gorica SI‐5000 Slovenia; ^3^ School of New Energy Materials and Chemistry Leshan Normal University Leshan Sichuan 614000 China; ^4^ Department of Chemistry Ben Gurion University of the Negev Beer Sheva 84105 Israel; ^5^ University of Strasbourg CNRS ISIS UMR 7006, 8 Allée Gaspard Monge Strasbourg 67000 France

**Keywords:** crystallization mechanisms, dilution‐induced kinetic trapping, phase transformations, photogating effect, phototransistors, tunable CsPbBr_3_/Cs_4_PbBr_6_ quantum dots

## Abstract

Precise control of quantum structures in hybrid nanocrystals requires advancements in scientific methodologies. Here, on the design of tunable CsPbBr_3_/Cs_4_PbBr_6_ quantum dots are reported by developing a unique discrete phase transformation approach in Cs_4_PbBr_6_ nanocrystals. Unlike conventional hybrid systems that emit solely in the green region, this current strategy produces adjustable luminescence in the blue (450 nm), cyan (480 nm), and green (510 nm) regions with high photoluminescence quantum yields up to 45%, 60%, and 85%, respectively. Concentration‐dependent studies reveal that phase transformation mechanisms and the factors that drive CsBr removal occur at lower dilutions while the dissolution–recrystallization process dominates at higher dilutions. When the polymer‐CsPbBr_3_/Cs_4_PbBr_6_ integrated into a field‐effected transistor the resulting phototransistors featured enhanced photosensitivity exceeding 10^5^, being the highest reported for an *n*‐type phototransistor, while maintaining good transistor performances as compared to devices consisting of polymer‐CsPbBr_3_ NCs.

## Graphical abstract

This study demonstrates a kinetically controlled discrete phase transformation process that converts non‐luminescent Cs_4_PbBr_6_ nanocrystals into hybrid Cs_4_PbBr_6_/CsPbBr_3_ quantum structures emitting blue (430 nm), cyan (480 nm), and green (510 nm) light. The resulting hybrid QDs can be integrated with organic semiconducting polymers to achieve high‐performance n‐type field‐effect phototransistors.

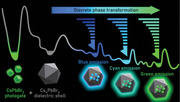

## 1 Introduction

Advancements in tunable light‐emitting CsPbBr_3_ perovskite quantum structures have made significant contributions to the field of optoelectronic devices,^[^ ^1^, ^2^ ^]^ driving major progress in energy harvesting,^[^ ^3^ ^]^ lighting,^[^ ^4^ ^]^ and photogating applications.^[^ ^5^ ^]^ To achieve the desired quantization, the size dimensions must be below 7 nm, corresponding to atomic‐level nanostructures such as nanowires, nanoplatelets, or nanosheets.^[^ ^6^ ^]^ However, challenges arise from rapid reaction rates, self‐aggregation, and the loss of optical properties over time^[^ ^7^ ^]^ Current methods for stabilizing perovskites such as core‐shell structuration^[^ ^8^ ^]^ or unique ligand functionalization^[^ ^9^ ^]^ have not been successful in tailoring quantum structures. Therefore, the development of heterostructures capable of serving as better scaffolds for accommodating these perovskite quantum structures is crucial. Once such class of heterostructure materials is the mixed‐multi‐dimensional (0D/3D) CsPbBr_3_/Cs_4_PbBr_6_ hybrid crystals which operate in the non‐quantized regime according to previous reports in the literature.^[^ ^10^ ^]^ The alignment compatibility between the two crystal systems CsPbBr_3_ NC with the lattice of Cs_4_PbBr_6_ results in enhanced passivation of the surfaces without inducing significant strain on the NC. This alignment has positive implications for the quality of a hybrid crystal and resulting optoelectronic properties.^[^ ^11^ ^]^ Hence, the progression of CsPbBr_3_/Cs_4_PbBr_6_ perovskites would benefit from the development of their quantum‐confined architectures, an area that remains largely unexplored.

In this study, we present a simple post‐synthetic transformation method to convert rhombohedral Cs_4_PbBr_6_ nanocrystals (NCs) into tunable luminescent CsPbBr_3_/Cs_4_PbBr_6_ hybrid QDs. Previous studies demonstrated a one‐step phase transformation of Cs_4_PbBr_6_ NCs to CsPbBr_3_ NCs through chemical^[^ ^12^ ^]^ and physical stimuli.^[^ ^13^, ^14^ ^]^ Additionally, the direct 0D to 3D phase transformation kinetics pose difficulties in understanding the mechanistic aspects.^[^ ^15^ ^]^ There are conflicting theories have been demonstrated to explain this transformation, which includes the removal of CsBr from the lattice,^[^ ^12^, ^16^ ^]^ solvent‐induced dipole moment changes,^[^ ^17^ ^]^ or complete dissolution of Cs_4_PbBr_6_ NCs followed by recrystallization into the CsPbBr_3_ phase.^[^ ^10^, ^18^ ^]^ In contrast to these to these one‐step phase transformation protocols, here, we introduce discrete phase transformation that allows seizure of a new class of intermediate states via well‐defined surface modification, and explore alternative strategies for composition control. Unfortunately, a great obstacle here is a poor understanding of 0D to 3D phase transformation processes because of their rapid reaction kinetics.^[^ ^10a^ ^]^ Furthermore, detailed information such as transient optical properties, reaction feasibility with respect to precursor concentration, and compositional evaluation is crucial for a comprehensive understanding of this reaction process. So far, the traditional synthetic reports on non‐quantized CsPbBr_3_/Cs_4_PbBr_6_ hybrid structures have predominantly focused on generating green‐emitting species, suggesting that the CsPbBr_3_ NCs inclusions within the Cs_4_PbBr_6_ matrix extend beyond the quantum‐confined region.^[^ ^19^ ^]^ Consequently, establishing compositions other than the green‐emitting CsPbBr_3_/Cs_4_PbBr_6_ NCs, tuning the sizes of CsPbBr_3_ inclusions within the quantum confinement regimes in CsPbBr_3_/Cs_4_PbBr_6_ QDs poses a significant challenge.^[^ ^20^ ^]^ It is important to note that the pure Cs_4_PbBr_6_ crystals, without halogen defects^[^ ^21^ ^]^ or perovskite inclusions,^[^ ^22^ ^]^ exhibit a non‐luminescent or weak blue emission due to high excitation energy and domination of nonradiative recombination^[^ ^10^, ^12^, ^23^ ^]^ Recent studies by Chen et al. have obtained tunable emissive properties in the blue, cyan, and green region in bulk Cs_4_PbBr_6_ crystals by discreate crystallization process through thermodynamic control.^[^ ^24^ ^]^ However, replicating such structures at the nanoscale remains challenging due to the lack of controlled synthetic methods. Overcoming these synthetic challenges not only holds the potential for constructing novel hybrid structures within the quantum‐confined region but also expands the utility of this emerging class of hybrid perovskites, thereby pushing the boundaries of perovskite research.

To address the fundamentals in phase transformation and crystal growth, we have developed a dilution‐induced, kinetically controlled discrete phase transformation approach that enables the trapping of metastable species with adjustable luminescence across the blue, cyan, and green‐emitting range (430–520 nm) in CsPbBr_3_/Cs_4_PbBr_6_ QDs. This approach differs from the fabrication of conventional non‐quantized hybrid systems that emit solely in the green region. The optical, spectroscopic, and electrical properties of these nanostructures were characterized to confirm the presence of CsPbBr_3_ inclusions in Cs_4_PbBr_6_. To the best of our knowledge, such precise control of CsPbBr_3_ quantum confinement in Cs_4_PbBr_6_ NCs remains unique among this class of hybrid perovskites. The process begins with non‐luminescent Cs_4_PbBr_6_ NC solutions, which undergo controlled phase transfer at different dilutions upon heating at 70 °C leading to the formation of CsPbBr_3_/Cs_4_PbBr_6_ QDs. Next. systematic investigations into this OD to 3D discrete phase transformation approach have revealed that this process involves the combination of several mechanistic aspects reported individually in the literature.^[^ ^10^, ^12^, ^18^, ^25^ ^]^ At specific dilutions, two processes dominate, i) the controlled leaching of CsBr from the Cs_4_PbBr_6_ crystal lattice leading to the formation of hybrid CsPbBr_3_/Cs_4_PbBr_6_ QDs, and ii) the complete self‐dissolution followed by recrystallization leading to formation of pure CsPbBr_3_ QDs. In the present study, we observed the mechanistic path of “CsBr leaching” being dominant at higher concentrations and “self‐dissolution” being favored at extreme dilutions of the Cs_4_PbBr_6_ NCs. Based on this binary reciprocal trajectory, we postulate that the phase transformation process in 0D Cs_4_PbBr_6_ NCs enables the precise control over the formation of either “hybrid CsPbBr_3_/Cs_4_PbBr_6_ QDs” or “pure CsPbBr_3_ QDs”. The obtained CsPbBr_3_/Cs_4_PbBr_6_ QDs were blended with organic semiconductors and utilized as an active layer into photo‐field effect transistors (photo‐FETs). Owing to the strong photogating capabilities of the CsPbBr_3_ QDs and the insulating nature of Cs_4_PbBr_6_, the hybrid photoFETs featured high photosensitivity combined with robust field‐effect transistor operation.

## 2 Results and Discussion

To investigate the kinetics of phase transformation in 0D Cs_4_PbBr_6_ NCs and their transition to quantum‐confined hybrid perovskite phases, a series of solution‐based studies were conducted, coupled with real‐time optical and microscopy tracking. The experimental conditions were systematically designed by varying the concentrations of colloidal Cs_4_PbBr_6_ NCs in a suitable solvent (octadecane (ODE) or hexane) and the reaction temperature was gradually raised from room temperature (RT) to higher temperatures (70–140 °C) to achieve the desired goals. The influence of temperature on the NC's concentration was emphasized, providing crucial insights for designing optimized conditions to create precisely tunable emitting CsPbBr_3_/Cs_4_PbBr_6_ QDs.

Throughout this phase transformation studies, monodisperse non‐emissive Cs_4_PbBr_6_ NCs with a size of 11.00 ± 0.04 nm were utilized (Figure S1, Supporting Information). These NCs were prepared by the regular hot‐injection (HI) method employed for the synthesis of perovskite NCs, with slight modifications in terms of ligand concentrations.^[^ ^10a^ ^]^ These NCs exhibit strong optical absorption at 314 nm (Figure S2, Supporting Information) attributed to the ^1^S_0_ → ^3^P_1_ transition of Pb^2+^ centers of Cs_4_PbBr_6_ NCs,^[^ ^26^ ^]^ and exhibits higher absorption strength, significantly higher than its 3D perovskite phase.^[^ ^27^ ^]^ The as synthesized NCs appears in a turbid white solution (inset in Figure S2, Supporting Information), which became transparent upon higher dilutions. The as‐synthesized Cs_4_PbBr_6_ NCs solution (original solution) without any dilution was labeled as “O”. In contrast, the reaction mixture diluted two times or three times and so on, compared to the undiluted condition were labeled as “O_2d_” or “O_3d_,” respectively. The peak intensity at 314 nm in the absorption spectra recorded from different dilutions of Cs_4_PbBr_6_ NCs reveals a variation in the molar absorption coefficient, which was decreased with the increasing NC dilution. This behavior differs from the traditional gold NCs, where the molar absorption coefficient is generally known to remain constant upon dilution (as shown in Figure S3, Supporting Information). These results strongly suggest that dilution induces surface‐level alterations in Cs_4_PbBr_6_ NCs, potentially affecting the composition, structure, or chemical properties that can impact the kinetics and mechanistic paths of phase transformation process, which will be further explored in subsequent sections.

### 2.1 Effect of Dilution on Transformation Temperature of Cs_4_PbBr_6_ NCs

**Figure 1:**
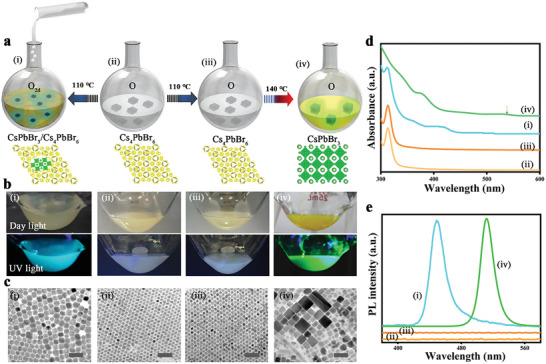
Concentration controlled phase transformation process in Cs_4_PbBr_6_ NCs. a) Schematic representation of the phase transformation process conducted at two different dilutions of Cs_4_PbBr_6_ NCs: i) O_2d_‐Cs_4_PbBr_6_ NCs obtained by heating pure Cs_4_PbBr_6_ NCs at 110 °C, ii) O‐Cs_4_PbBr_6_ NCs, iii) O‐Cs_4_PbBr_6_ NCs heating at 110 °C, and iv) O‐Cs_4_PbBr_6_ NCs further heated at 140 °C. b) Respective photographs of the reaction solutions presented in scheme (a), collected at daylight and UV light. c) TEM images showcasing the morphology of the respective i) CsPbBr_3_/Cs_4_PbBr_6_ QDs, ii,iii) Cs_4_PbBr_6_ NCs, and iv) CsPbBr_3_ NCs. The scale bars correspond to 50 nm. d,e) UV/vis absorption spectra (d) and photoluminescence spectra (e) of the corresponding solutions (i–iv).

To explore the relationship between inter‐NC interactions and the phase transition kinetics, a sequence of two reactions were carried out utilizing pristine Cs_4_PbBr_6_ NCs at different dilution conditions labeled as “O” and “O_2d_.” (**Figure** **1**a). The controlled parameter was the temperature which was gradually increased from RT to the designated reaction temperature to observe a noteworthy phase change within the NCs. The phase transformation is evidenced by the color changes evolving from colorless to a faint yellow, or characteristic strong emissions from the CsPbBr_3_ phase under UV light. Interestingly, the O_2d_‐Cs_4_PbBr_6_ NC solution exhibited a seamless shift from colorless to pale yellow upon reaching the temperature of 110 °C. In contrast, the O‐Cs_4_PbBr_6_ NC solution remained unchanged at this temperature. Furthermore, when exposed to UV light, solely the O_2d_ reaction shifted from non‐emissive to cyan emitting solutions, while the other solution did not display any luminescence (photographs are compared in Figure 1b‐i,iii). The lack of visible changes in the O‐solution indicates the importance of concentration which dictates the NC's surface for facilitating the transformation process. At 110 °C, the nature of these reactions, and their resulting crystal phase were studied by absorption spectra, photoluminescence (PL), X‐ray diffraction pattern (XRD), and transmission electron microscopy (TEM). TEM images revealed the size of the heated O_2d_‐Cs_4_PbBr_6_ NCs increased from 9 to 18 nm (Figure 1c‐i,ii), while the heated O‐Cs_4_PbBr_6_ NCs remained the same size as the initial Cs_4_PbBr_6_ NCs (Figure 1c‐iii). The absorption spectra of the heated O‐Cs_4_PbBr_6_ NCs were similar to those of the pristine NCs, showing a single peak at 314 nm (Figure 1d‐ii,iii). In contrast, the heated O_2d_‐Cs_4_PbBr_6_ NCs exhibited a strong absorption peak at 430 nm, along with a faint band at 320 nm, indicating the presence of a heterostructure (Figure 1d,i). The photoluminescence spectra of O_2d_‐NCs displayed an emission peak at 450 nm, likely due to the embedded CsPbBr_3_ QDs (Figure 1e‐i). On the other hand, the heated O‐Cs_4_PbBr_6_ NCs did not exhibit any photoluminescence, similar to the pristine NCs (Figure 1e‐ii,iii). XRD analysis of cyan emitting CsPbBr_3_/Cs_4_PbBr_6_ QDs shows the presence of Cs_4_PbBr_6_ NCs, but CsPbBr_3_ inclusions were not noticeable (Figure S4, Supporting Information) which is matching with the hybrid structures reported previously.^[^ ^12a^ ^]^ Majorly, this XRD, as well as TEM study, confirms that this cyan emission is originating from the hybrid structures, not from the individual CsPbBr_3_ NCs.

Furthermore, we explored the phase transformation temperature for the O‐Cs_4_PbBr_6_ NCs via raising the temperature gradually beyond 110 °C. A phase transition process on these viscous dispersions occurred at 140 °C, accompanied by a color change from turbid white to yellow green. The absorption spectra of the resulting sample resembled those of pure CsPbBr_3_ NCs, exhibiting a band at 510 nm and the absence of the 314 nm peak associated with Cs_4_PbBr_6_ (Figure 1d‐iv). This sample also displayed a strong green emission at 512 nm (Figure 1d‐iv). TEM images of this solution show the presence of polydisperse CsPbBr_3_ NCs with cubic morphology of sizes ≈30–60 nm (Figure 1c‐iv). The growth toward larger CsPbBr_3_ NCs resulted from the oriented self‐assembly of NCs governed by the phase transformation that occurred at higher temperature in these viscous conditions.^[^ ^28^ ^]^ It is worth comparing this with a typical HI method where 110–170 °C is considered as an ideal temperature for producing nearly monodisperse CsPbBr_3_ NCs of size ≈5–20 nm.^[^ ^29^ ^]^ However, under the same concentration conditions as the HI method, here, the phase transformation from 0D to 3D CsPbBr_3_NCs occurs only beyond 135 °C. Based on our previous^[^ ^10a^ ^]^ and other groups^[^ ^12^, ^30^ ^]^ reports, it is known that the Cs_4_PbBr_6_ phase could be kinetically stabilized from CsPbBr_3_ by the extraction of PbBr_2_ through the chelating molecules at RT. In our study, the colloidal Cs_4_PbBr_6_ NCs were also readily stabilized due to the presence of higher concentrations of PbBr_2_‐chelated molecules (ionic OA^−^ and Olam^+^). But for the reversible reaction, i.e., from Cs_4_PbBr_6_ to CsPbBr_3_ NCs, there are two possibilities; i) the release of CsBr into the polar environment, or ii) the incorporation of PbBr_2_ into the 0D NC lattices. To trigger either of these processes to occur in our case, one should perturb the PbBr_2_‐chelated molecules causing the release of perovskite formation precursors (Pb^2+^ and Br^−^). Consequently, the disruption of ionic species and the subsequent liberation of PbBr_2_ precursors may demand a higher energy input in comparison to the conventional HI method. This observation paves the way for the generation of novel perovskite materials by facilitating controlled phase transformations through the interplay between the reaction temperature and concentration of NCs and chelated molecules.

### 2.2 Dilution‐Based Kinetic Control: Tunable Emission in CsPbBr_3_/Cs_4_PbBr_6_ QDs

**Figure 2:**
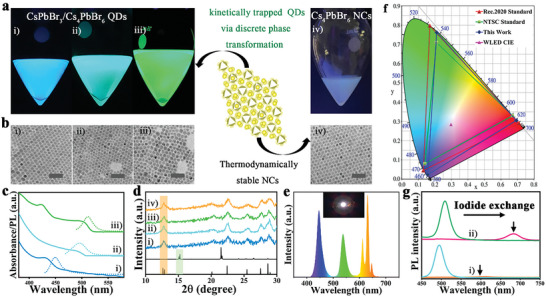
Tunable CsPbBr_3_/Cs_4_PbBr_6_ QDs through the discrete phase transformation process, anion exchange process, and exploring the WLED properties. a) Photographs of the dilution‐based kinetically trapped i) blue, ii) cyan, and iii) green emitting CsPbBr_3_/Cs_4_PbBr_6_ QDs, and iv) the thermodynamically stable O‐Cs_4_PbBr_6_ NCs. These photographs are recorded under UV light. b) TEM images of the respective solutions (i–iv). The scale bars correspond to 50 nm. c) UV/vis absorption spectra and PL spectra of the blue (i), (ii), and green (iii) emitting CsPbBr_3_/Cs_4_PbBr_6_ QDs. d) XRD patterns of all the above‐mentioned NCs (i–iv). e) Electroluminescence spectrum of the WLED device fabricated using green emitting CsPbBr_3_/Cs_4_PbBr_6_ QDs, inset is the photograph of the device and its CIE coordinates displayed in (f). g) PL changes up on the Br^−^ to I^−^ exchange in cyan i) and green ii) spectra emitting CsPbBr_3_/Cs_4_PbBr_6_ QDs. Arrow marks indicate the final PL emission from CsPbBr_3‐x_I_x_/Cs_4_PbBr_6_ QDs after completion of Br^−^ to I^−^ exchange.

Based on the previous experiments concerning phase transformation in Cs_4_PbBr_6_ NCs, it has been observed that the transformation process of Cs_4_PbBr_6_ NCs could be kinetically controlled through dilution rather than by thermodynamic control. As a result, we introduced the dilution‐based kinetic control approach by adjusting the level of dilution in a desired reaction for designing a specific composition of hybrid CsPbBr_3_/Cs_4_PbBr_6_ QDs. Toward this end, a series of phase transformation reactions conducted by changing the concentration of NCs by diluting the O‐Cs_4_PbBr_6_ NCs with n‐hexane (O_3d_, O_5d_, and O_10d_) was followed by heating at 70 °C as can be seen in **Figure** **2**a and Figures S5 and S6 (Supporting Information).

Interestingly, the initial non‐luminescent Cs_4_PbBr_6_ NCs (Figure 2a‐iv) evolve into hybrid CsPbBr_3_/Cs_4_PbBr_6_ QDs exhibiting a gradual shift in their emitting properties from blue, cyan, and green while diluting the reaction solutions (photographs in Figure 2a). Previous studies by Chen et al. have reported similar emission colors in bulk Cs_4_PbBr_6_ crystals, ranging from blue to cyan and green, due to the presence of differently sized CsPbBr_3_ inclusions.^[^ ^24^ ^]^ So far, such controlled emission in these Cs_4_PbBr_6_ NCs has not been established. These three (O_3d_ O_5d_ and O_10d_) phase transformation reactions, in their absorption spectra show the presence of Cs_4_PbBr_6_ matrix from their characteristic 313 nm peak along with the other characteristic peaks at 430 and 480, and 500 nm, respectively, indicating the presence of hybrid structures (Figure 2c). The PL spectra of these QDs are Stokes‐shifted with respect to the optical absorption spectra. The PL emissions of these reactions were located at 450, 485, and 510 nm (Figure 2c). The PLQY for the green, cyan, and blue‐emitting hybrid QDs synthesized in this study were 85%, 60%, and 45%, respectively. The PLQY of the green‐emitting hybrid systems was notably higher, yet still slightly lower than that of best values reported in literature for surface‐treated hybrid structures. It is worth mentioning that the PLQY of CsPbBr_3_/Cs_4_PbBr_6_ hybrid structures is influenced by the size of the host material, as indicated in the literature. PLQY values ranging from 70% to near unity^[^ ^19^, ^31^, ^32^ ^]^ for surface passivated structures while single crystals and hybrid NCs in the range of 30–45%.^[^ ^19^, ^33^ ^]^

These QDs were precipitated by the addition of methyl acetate (MA) and used for further characterization or WLED fabrication. Figure 2d shows the powder XRD patterns of the resulting QDs obtained from these samples. The characteristic diffraction peaks of Cs_4_PbBr_6_ NCs at ≈12° were found across all the samples, indicating that these samples contained Cs_4_PbBr_6_ NCs. Next, we fabricated a WLED by using CsPbBr_3_/Cs_4_PbBr_6_ QDs and K_2_SiF_6_:Mn^4+^ as green and red‐emitting phosphors, respectively, and a blue chip as the excitation source. The result of electroluminescence (EL) spectrum of the WLED device is shown in Figure 2e. The EL spectrum is composed of three emission bands: blue, green, and red, which belong to the blue LED chip, our samples phosphor, and KSF phosphor, respectively. The constructed WLED device exhibited the CIE color coordinates (0.3054,0.3043). Figure 2f, a color temperature of 8143 K, an efficiency 20.31 lm W^−1^, the obtained color gamut of the assembled backlight unit is compared with the NTSC and Rec.2020 standards, this work color gamut can cover ≈120.0% and 89.6% of the NTSC and Rec.2020, respectively. We also tested the color stability of the prepared WLED devices under different driving currents, as shown in Figure S7 (Supporting Information). With the increase of drive current, the emission intensity of Cs_4_PbBr_6_ increases, and the CIE of WLED device changes little. However, we notice that the efficiency is poor when compared to the previous bulk hybrid crystals whose efficiency is typically in the range of 50–150 lm W^−1^.^[^ ^19^, ^24^ ^]^ This is due to the reduction in PLQY of such hybrid materials in solid state with decrease in their crystal sizes. In contrast to the pure CsPbBr_3_ perovskites, where bulk crystal shows poor emission whereas bulk or micron‐sized hybrid structures exhibit strong emission in their solid state.^[^ ^27^ ^]^ Figure 2b displays the TEM analysis of these hybrid NCs which are comparable to that of the pure Cs_4_PbBr_6_ NCs exhibiting rhombohedral or pseudospherical shapes. The pure NCs possess a diameter of ≈10 nm, while the blue, cyan and green hybrid QDs exhibit diameters of ca. 13 nm, respectively. We have not found any CsPbBr_3_‐related phases (nanocubes or nanowires) in this TEM analysis also indicating these species are of hybrid QDs.

The formation of quantized hybrid QDs in this phase transformation process is further confirmed by the anion‐exchange method, where iodide precursor was introduced into these systems and monitored their exchange kinetics by in situ PL changes (Figures S8 and S9, Supporting Information). A red shift in the PL emission stemming from the formation of CsPbBr_3‐x_I_x_ from CsPbBr_3_ inclusions in Cs_4_PbBr_6_ is expected. Unlike in the case of the pure CsPbBr_3_ NCs, the iodide exchange reaction does not occur in the blue‐emitting CsPbBr_3_/Cs_4_PbBr_6_ QDs, as the PL emission quenching upon the addition of iodide precursor, hence does not lead the formation of CsPbBr_3‐x_I_x_/Cs_4_PbBr_6_ QDs. The iodide exchange in the case of cyan emitting CsPbBr_3_/Cs_4_PbBr_6_ QDs underwent only a limited amount of iodide‐exchange evidenced by the slight shift from 480 to 600 nm (Figure 2g‐i). In addition, the intensity was drastically decreased, with no future shift in the PL position.  The iodide exchange on the green hybrid structure too shows a drastic quenching in the PL emission while the PL wavelength shifted to 680 nm from 520 nm (Figure 2g‐ii). The integrated intensities of the PL spectra of each exchanged QDs drastically quenched compared to parent samples which is in contrast to the pristine CsPbBr_3_ NCs. At the similar exchange synthetic conditions, CsPbBr_3_ NCs demonstrate more proficient exchange kinetics and substantially enhanced PL intensities.^[^ ^34^ ^]^ The inefficiency of this Br^−^ to I^−^ exchange in hybrid structures is attributed to the fact that it occurs only at the outermost layers of the material due the presence of a protective shell around CsPbBr_3_ NCs.

**Figure 3:**
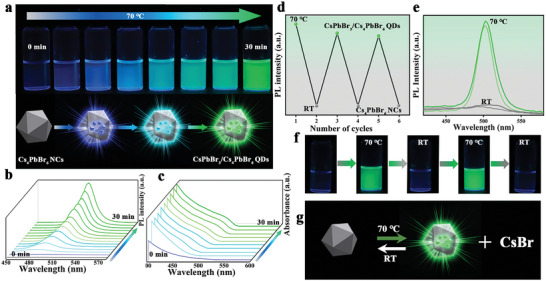
Controlled growth of non‐luminescent Cs_4_PbBr_6_ to CsPbBr_3_/Cs_4_PbBr_6_ QDs and their reversibility. a) Photographs of the colloidal solutions collected under UV light, showing the progression of non‐luminescent Cs_4_PbBr_6_ NCs to green‐emitting CsPbBr_3_/Cs_4_PbBr_6_ QDs in hexane; schematic representation of this transformation is provided at the bottom of (a). b,c) Time‐dependent changes in PL spectra (b) and UV/vis absorption spectra (c) during the transformation of Cs_4_PbBr_6_ NCs to green‐emitting CsPbBr_3_/Cs_4_PbBr_6_ QDs. d–g) The formed green emitting CsPbBr_3_/Cs_4_PbBr_6_ QDs turned back to non‐luminescent Cs_4_PbBr_6_ NCs and this process is reversible at two different temperatures (RT and 70 °C) (schematically represented in g). Reversible cycles (d) among QDs to NCs, and respective PL spectra (e) and photographs under UV light (f) are presented.

**Figure** **3**a–c shows the evaluation of optical properties during the phase transformation from O_10d_‐Cs_4_PbBr_6_ NC toward the hybridization in green emissive CsPbBr_3_/Cs_4_PbBr_6_ QDs at 70 °C. The initial absorption spectra with a prominent band at 314 nm, corresponding to the Cs_4_PbBr_6_ NCs remained consistent for the first 10 minutes at constant heating under ambient conditions. After ≈12 min of heating, a new absorption band emerged at 450 nm which coexisted with the original band at 314 nm, indicating the simultaneous presence of both phases. Under continuous heating for the next 30 min, a progressive redshift of the wavelength from 460 to 515 nm was observed.  Simultaneously, the PL emission changed from a non‐emissive state to green emissive species, with PL emissions occurring at various wavelengths: 460 nm (12 min), 490 nm (18 min), 500 nm (20 min), and 510 nm (25 min), ultimately reaching 520 nm. Interestingly, the intensity of the PL emission gradually increased during these wavelength changes due to the increased quantum yield. Subsequently, there were no further alterations in the absorption and emission positions upon continuous heating for the next hour. Similarly, the emergence of cyan CsPbBr_3_/Cs_4_PbBr_6_ QDs from O_5d_‐NCs was investigated through the time‐dependent absorption and PL and is presented in Figure S10 (Supporting Information). The absorption spectra at ≈10 min, a new absorption band appeared at 470 nm, alongside the initial absorption band at 314 nm. After 20 min of heating, the absorption band shifted to 485 nm, and the PL emission occurred at 490 nm. Remarkably, these values remained stable even after continuous heating for an additional 30 min, indicating the cyan emitting CsPbBr_3_/Cs_4_PbBr_6_ QDs are kinetically stable at this dilution.

Unlike the pure CsPbBr_3_ NCs, these solutions of hybrid structures turned back to the initial Cs_4_PbBr_6_ structures within a few hours while standing in ambient conditions (Figure 3d–g). This also indicates that these QDs are composed of hybrid perovskite structures and the continuous incorporation of CsBr or removal of PbBr_2_ from the lattice leads the structures of the original non‐emissive Cs_4_PbBr_6_ NCs. At the same time intervals, the fully formed CsPbBr_3_ NCs remained stable for several days without a change in their PL intensity. The reversible transformation reaction also followed a systematic backward trend, i.e., the green hybrid system first transformed to cyan, followed by blue, and finally to non‐emissive species. The reversed non‐emissive species again underwent forward transformation from non‐emissive to emissive upon being subjected to heating (Figure 3f). We studied such reversibility on green emissive samples for up to three cycles (Figure 3d,e). This kind of reversible transformation was also seen in the case of cyan and blue emitting CsPbBr_3/_Cs_4_PbBr_6_ QDs (Figure S11, Supporting Information). However, the time required for reversible transformation of hybrid perovskites to non‐emissive Cs_4_PbBr_6_ NCs varied, and was in the following order, blue hybrid perovskites (1 h) < cyan hybrid perovskites (6 h) < green hybrid perovskites (15 h). This trend is directly proportional to the amount of CsBr required to remove from the hybrid perovskites to reach the pure 0D structures. This indicates that the quantitative removal of PbBr_2_ or addition of CsBr in the hybrid perovskite lattices leads to the pure Cs_4_PbBr_6_ NCs.

### 2.3 Dilution‐Triggered Transformation of Cs_4_PbBr_6_ NCs to CsPbBr_3_ NCs: Thermodynamics and Kinetics

**Figure 4:**
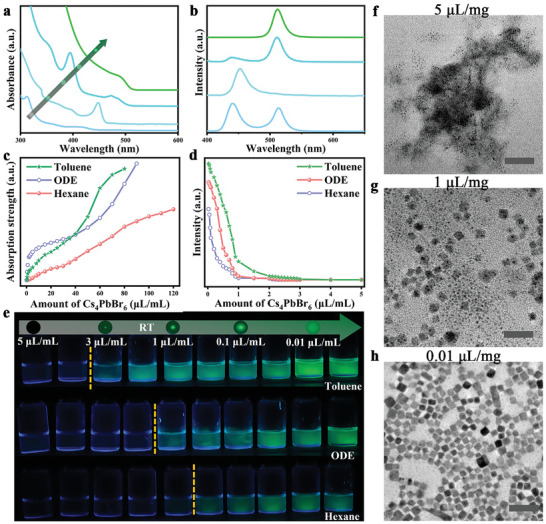
Spontaneous transformation of Cs_4_PbBr_6_ NCs to CsPbBr_3_ NCs in different solvents at extreme dilutions and mechanistic details. a,b) UV/vis absorption spectra (a) and PL spectra (b) of Cs_4_PbBr_6_ NCs at various dilutions in hexane. c,d,e) The changes in the optical properties of Cs_4_PbBr_6_ NCs as a function of different dilutions in the three different solvents. The change in the absorption strength at 314 nm (c), the change in the PL intensities (d), and respective photographs under UV light (e). The dotted lines represent the emergence of blue range of emission under UV light which varies depend on the type of solvent. NC solutions are at different concentrations in the three different solvents. f–h) TEM images collected at the intermediated stages of spontaneous transformation from Cs_4_PbBr_6_ NCs to CsPbBr_3_ NCs. The scale bar corresponds to 50 nm.

We found that the feasibility of the phase transformation in Cs_4_PbBr_6_ NCs into respective CsPbBr_3_/Cs_4_PbBr_6_ QDs without using any external chemical stimuli is highly influenced by the thermodynamic barrier. The above findings pointed out that this thermodynamic activation energy could be manipulated through the concentration of NCs which can be regulated manually and is inversely proportional to the NC's dilution. If this is the case, one can tune this transformation spontaneously at RT toward well‐defined phase pure quantum confined CsPbBr_3_ structures at optimized dilutions. To investigate this phenomenon, we conducted a range of progressive dilutions of NCs spanning from moderate to significantly higher dilutions (O_5d_ to O_1000d_, corresponding to the concentrations of 5 to 0.025 µL mL^−1^) using three distinct and independent solvent systems, namely toluene, ODE, and hexane. **Figure** **4**a shows the absorption spectra of these NC solutions at four different dilutions showing a systematic shift in the exciton peak position from 420, 450, 480, and 520 nm, respectively. Accordingly, the PL intensity of these NCs also shifted from 430 to 520 nm. The emergence of PL emission as a function of dilution in all these solvents is portrayed in Figures S12–S14 (Supporting Information). The absorption peak intensity at 314 nm was only present for the blue emitting perovskite CsPbBr_3_ sample (Figure 4a), whereas the corresponding peak is absent in the next samples showing the presence of pure CsPbBr_3_ NCs. XRD data further confirms the presence of mixed phases (OD and 3D) in the blue emitting sample (Figure S15, Supporting Information) and the presence of pure orthorhombic CsPbBr_3_ NCs in later cases (Figure S16, Supporting Information). Figure 4c,d presents a change in absorption peak intensities (absorption strength) at 314 nm and the evaluation of PL emission across 30 systematic dilutions of Cs_4_PbBr_6_ NC in various solvents (toluene, ODE, and hexane) at RT (Figure S17, Supporting Information). Based on the absorption spectra, dilution versus and the absorption peak intensity displays nearly linear reduction in the absorption strength and finally reaches to near zero at a certain threshold ≈3 µL mL^−1^ (Figure 4c,d). Interestingly, this dilution leads toward a significant change in the optical properties, associated with the disappearance of absorption at 313 nm and the appearance of new bandgap energies as well as emergence of PL spectra readily tunable over the blue‐green region in ca. 30–60 s.  (Figure 4b; Figure S18, Supporting Information). Notably, the raise of PL emission at the expense of absorption at 314 nm indicating the dissolution of Cs_4_PbBr_6_ NCs and recrystallization into CsPbBr_3_ quantum structures. The PL intensity profile shows a drastic enhancement at increased dilutions. The solutions under UV light irradiation also reflect similar results showing the transformation from non‐luminescent Cs_4_PbBr_6_ NCs to luminescent blue, cyan, and green‐emitting single‐phase CsPbBr_3_ QDs (photographs presented in Figure 4e). These dilution‐mediated changes in absorption and PL emissions are remained consistent across toluene, ODE, and hexane solvents, albeit with slight differences in intensities. For instance, hexane began to exhibit emissions in the blue region at higher dilutions, while toluene reached this stage at lower dilutions (represented by dotted lines in Figure 4e). The required NC's dilutions to initiate the spontaneous 0D to 3D transformations in colloidal solutions followed the order: hexane (O_15000d_) <ODE (O_3000d_) < toluene (O_1000d_). The polarity of these solvents also followed a similar order: toluene > ODE > hexane, indicating along the influence of dilution, there is a correlation between the phase transformation trend and solvent polarity.^[^ ^35^ ^]^

In addition, the TEM analysis of this transformation in hexane at different dissolutions were studied, the trend showed that the Cs_4_PbBr_6_ NCs dissolute to CsPbBr_3_ nanoclusters followed by a systematic growth toward CsPbBr_3_ nanoplatelets and nanocubes during this process (Figure 4f–h; Figure S19, Supporting Information). The intermediate phases composed of partially dissolute‐hybrid structures, lamellar structures along with CsPbBr_3_ nanoclusters, nanoplatelets, also noticed in TEM analysis (Figure S20, Supporting Information).

**Figure 5:**
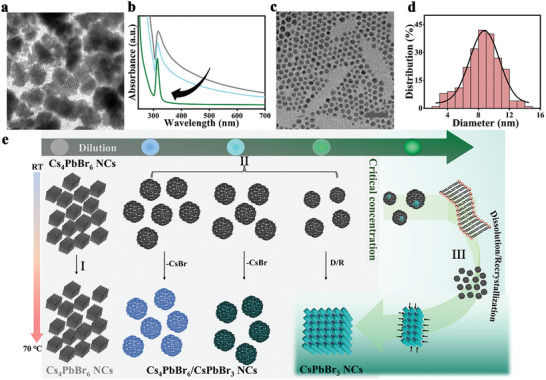
a) TEM image of Cs_4_PbBr_6_ NCs at higher concentrations show the presence self‐aggregated O‐Cs_4_PbBr_6_ NCs. The scale bar corresponds to 200 nm. b) The changes in the UV/vis absorption spectra of the colloidal O‐Cs_4_PbBr_6_ NCs as a function of dilution. c,d) TEM image and particle size distributions represents the reduction in the average size of Cs_4_PbBr_6_ NCs at higher dilutions (O_10d_ in hexane). The scale bar corresponds to 50 nm. e) Schematic representation of the mechanistic insights into the discrete phase transformation process in Cs_4_PbBr_6_ NCs, concluded based on various systematic concentration and temperature dependent studies.

Based on our observations and previous reports^[^ ^10^, ^12^ ^]^ we propose this whole process of transformation as shown in the **Figure** **5**a–d. Note that the facets of the rhombohedral Cs_4_PbBr_6_ NC phase is considered to be neutral because of the passivated oleate–ammonium complex could stabilize the neutral‐charged surface composed of negatively charged PbBr_6_ octahedra partially balanced by in‐plane Cs^+^ ions.^[^ ^25^ ^]^ Hence, at higher concentrations, the Cs_4_PbBr_6_ NCs in solutions tended to self‐aggregate as evidenced by the visible appearance of turbid‐like solutions in the as synthesized NC solution. Moreover, at this concentration the absorption peak position was slightly red‐shifted to 317 nm (gray line in Figure 5b) with increased peak width along with substantially high scattering part compared to the isolated NCs (green line in Figure 5b). The scattering part and the peak width were gradually reduced upon additional dilution and the peak position was back original (314 nm). TEM analysis of the concentrated colloidal solution revealed the presence of self‐assembled structures of sizes in the range of few hundred nanometers (Figure 5a). In contrast, at dilute conditions, the TEM images showed separate individual NCs (Figure 5c). This indicates that the spontaneous assembly of the individual NCs to ordered structures occurred at higher concentrations, which lowers the free energy through the decrease in the surface energy. Thus, the highly‐organized self‐assembled NCs suspensions are thermodynamically stable. Furthermore, the close proximity of the NCs restricts the available reaction space, which prevents the mass transfer from individual NCs to the surrounding environment. As a result, the transformation process was kinetically hindered (Figure 5e‐i). Dilution caused the NCs to separate from their self‐assembled state, resulting in increased surface energy and allowing them to interact with the ligands (ionic oleylamine and oleic acid). Only upon heating, this condition favored the release of CsBr from the Cs_4_PbB_6_ NCs leading to the formation of CsPbBr_3_ nano‐inclusions within the Cs_4_PbB_6_ matrix. The extent of CsBr loss could influence the formation of CsPbBr_3_ inclusions within the Cs_4_PbB_6_ matrix, which was determined by local NCs concentrations.^[^ ^30^ ^]^ In this scenario, the quantized emissions (blue, cyan, green) originated from the CsPbBr_3_/Cs_4_PbBr_6_ QDs structures (Figure 5e‐ii).

The situation of the NCs in solution upon gradual increase in dilution was studied by TEM and dynamic light scattering (DLS). Figures S21–S23 (Supporting Information) show the separation of NCs from the rhombohedral‐type self‐aggregated crystals to the individual NCs via the hexagonal packing monolayered NCs. At increased dilution before the phase transformation the nanoparticles were smaller in size (8.7 ± 0.1 nm) compared to the original NCs (11 ± 0.1 nm) further confirming the surface and compositional modifications (Figure 5c,d). From these observations, at excessive dilutions (O_1000d_ or more), the dissociation of PbBr_2_‐ligands and increased ligand influences acted on individual NCs, leading to excessive release of CsBr and subsequent loss of Cs_4_PbBr_6_ NCs identity. This abrupt change in the system at higher dilutions ultimately resulted in the dissolution of Cs_4_PbBr_6_ NCs and their recrystallization into quantum‐confined CsPbBr_3_ NCs through well‐controlled crystal growth conditions at RT (Figure 5e‐iii).

## 3 Hybrid Organic/Inorganic Field‐Effect Phototransistors with CsPbBr_3_/Cs_4_PbBr_6_ QDs

So far, research on perovskite‐based photoFETs (3‐terminal devices that can be both operated electrically with a gate electrode and optically by illumination of the channel) has primarily concentrated on enhancing device performance in terms of photosensitivity (P) (where P = I_photocurrent_/I_dark_ with I_photocurrent_ = I_illumination_ − I_dark_) and photoresponsivity.^[^ ^36^ ^]^ In pursuit of optimizing higher P, previous studies have primarily leveraged gate control to induce a threshold voltage (V_Th_) shift, resulting in exceptionally high P values at specific gate voltages (V_GS_). While this approach has proven highly effective in reducing I_dark_ and elevating I_photocurrent_, it presents several challenges when it comes to integrating these devices into practical electronics applications.

First, the operation of these devices frequently demands high drain voltages often exceeding 10 volts. and typically employ interdigitated architectures to obtain easily measurable drain‐source current (I_DS_) values. Furthermore, for photoFETs to be integrated into photoresponsive circuits such as photoFET‐based inverter circuits,^[^ ^37^ ^]^ high FET performances in terms of mobility and I_on_/I_off_ and an adequate V_Th_ are required.^[^ ^37^ ^]^ As such, to achieve devices that can fully combine logic and photoresponse features, FET performances should not be sacrificed. Since these devices rely on the photogating effect, further improvements in terms of performance could be achieved by blending the perovskite and its derivatives into a semiconducting polymer layer.^[^ ^38^ ^]^ However, this strategy would result in an increase in unwanted interfacial effects, further lowering the transistor performances. These potentially unwanted interfacial effects are two. First, the interface trapping of charges by the perovskite will result in a decrease in electrical performance. Second, it is energetically favorable for charges to go from the perovskite into the semiconductor. Since the photogating effect relies on charges trapped in the perovskite to generate the electric field leading to an increase in current, it could be negatively affected by the application of a strong gate voltage. This could lead to a very strong decrease in the current when both “gates” are operating simultaneously. These effects can be mitigated by reducing the conductivity of the perovskite. Furthermore, a reduced conductivity of the perovskite would result in slightly reduced off currents at high concentrations of perovskites (and therefore slightly reduced dark currents), owing to the fact that pure perovskites pathways in the film would feature reduced charge transport.

**Figure 6:**
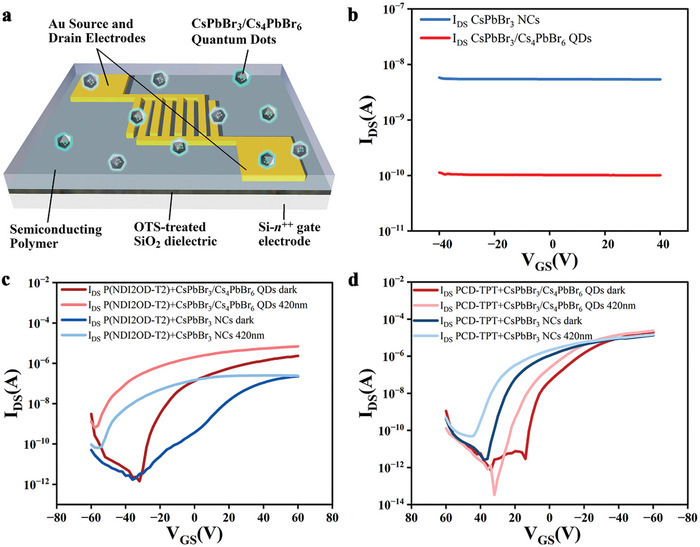
Electrical and optoelectrical characterization of the photoFETs. All curves for devices with L = 20 µm and W = 10 mm at VDS = −60 V. a) Structure of the photoFETs in bottom‐contact bottom‐gate architecture. b) Transfer curves of CsPbBr_3_ NCs and CsPbBr_3_/Cs_4_PbBr_6_ QDs films. c) Transfer curves of photoFETs based on the blends P(NDI2OD‐T2) + CsPbBr_3_ NCs and P(NDI2OD‐T2) + CsPbBr_3_/Cs_4_PbBr_6_ QDs, in the dark and under illumination at 420 nm. d) Transfer curves of photoFETs based on the blends PCD‐TPT + CsPbBr_3_ NCs and PCD‐TPT + CsPbBr_3_/Cs_4_PbBr_6_ QDs in the dark and under illumination at 420 nm.

To determine the conductivity and transistor operation of the green emitting CsPbBr_3_/Cs_4_PbBr_6_ QDs, bottom‐contact bottom‐gate field effect transistors (**Figure** **6**a) were fabricated with a ≈1 µm drop casted layer of perovskites in the channel. The I_DS_ current was measured as a function of the gate voltage and compared to CsPbBr_3_ NCs (Figure 6b). While neither device featured gate control, the currents of the transistor containing the hybrid perovskite were found to be 2 orders of magnitude lower as compared to the CsPbBr_3_ NCs containing device. The conductivity of the CsPbBr_3_ NCs and CsPbBr_3_/Cs_4_PbBr_6_ QDs films were found to be 4.2 × 10^−8^ and 1.718 × 10^−10^ S m^−1^ respectively. While both values were low, the conductivity of the CsPbBr_3_ NCs could still result in increased I_dark_ and reduced P, even in a thinner film. Illumination within the absorption range of the perovskites did not result in a major change in current (Figure S24, Supporting Information). Cyclic voltammetry measurements revealed no difference in energy levels between the two NCs (Figure S25a, Supporting Information) hence none of these perovskites should act as a trap for charges when blended with organic polymers, whether *p*‐ or *n*‐type (Figure S25b,c, Supporting Information).

Blends of NCs and organic semiconductors with equal wt% were deposited as active layer for photoFETs. These devices were characterized in the dark and under illumination at 420 nm (within the absorption range of the NCs). Two polymers were chosen for this experiment: poly ([N, N’‐bis(2‐octyldodecyl)‐naphthalene‐1,4,5,8‐bis(dicarboximide)−2,6‐diyl]‐alt‐5,5′‐(2,2′‐bithiophene)) (P(NDI2OD‐T2) and poly[4‐(4,4‐dihexadecyl‐4H‐cyclopenta[1,2‐b:5,4‐b’] dithiophen‐2‐yl)‐alt^[^ ^1^, ^2^, ^5^ ^]^ thiadiazolo[3,4‐c] pyridine] (PCD‐TPT), respectively *n*‐ and *p*‐type semiconductors. Considering the blended nature of the active layers in the photoFETs, P(NDI2OD‐T2) and PCD‐TPT were chosen due to their less ordered nature as compared to more commonly selected polymers such as poly(3‐hexylthiophene‐2,5‐diyl) and PDVT‐10.^[^ ^38^, ^39^, ^40^ ^]^ P(NDI2OD‐T2) has been exploited as it is one of the most commonly used n‐type polymers in previous studies on perovskite photogating.^[^ ^39^, ^40^ ^]^ Transfer curves of devices based on P(NDI2OD‐T2) + CsPbBr_3_ NCs, P(NDI2OD‐T2) + CsPbBr_3_/Cs_4_PbBr_6_ QDs, PCD‐TPT + CsPbBr_3_ NCs and PCD‐TPT + CsPbBr_3_/Cs_4_PbBr_6_ QDs were plotted in the dark and under illumination at 420 nm (Figure 6c,d; Figure S26, Supporting Information).

Each of the 4 blends resulted in devices in which the photogating effect was observable with currents much higher under illumination, at specific gate voltages, leading to P values over 10^3^. However, a strong increase in I_illuminated_ was observed in the case of the CsPbBr_3_/Cs_4_PbBr_6_ QDs containing devices. In particular, P(NDI2OD‐T2) + CsPbBr_3_/Cs_4_PbBr_6_ QDs photoFETs featured photosensitivity values is 1.85 × 10^5^, to our knowledge the highest observed value for an *n*‐type photoFET,^[^ ^41^ ^]^ and superior by two orders of magnitude as compared to previously reported bilayer devices based on P(NDI2OD‐T2) + CsPbBr_3_.^[^ ^38^, ^41^, ^42^ ^]^

This can be explained by two factors. First, the blending strategy is highly beneficial for the photogating effect since the amount of polymer in proximity to the NCs is strongly increased. Second, a reduction of the NCs pathways occurs since the polymers act as charge‐trapping elements leading to a reduced I_dark_. This effect is further compounded by the very low conductivity of the CsPbBr_3_/Cs_4_PbBr_6_ QDs. Furthermore, in the case of the blends with hybrid QDs, the effects of the photogating and the bottom gate of the device are less affected by each other as compared to the case of the CsPbBr_3_ NCs devices. The detrimental effect of the photogating on the transistor operation in the devices containing CsPbBr_3_ NCs is very strong leading to a large decrease in mobility upon illumination both in the case of P(NDI2OD‐T2) and PCD‐TPT containing photoFETs. However, this effect is not observed in CsPbBr_3_/Cs_4_PbBr_6_ QDs containing devices leading to a 13‐fold higher mobility for P(NDI2OD‐T2) containing photoFETs under illumination (Tables S1 and S2, Supporting Information). As compared to pure P(NDI2OD‐T2) devices, the transistor characteristics are mostly conserved in the dark with similar I_on_/I_off_ and mobility values reduced by one order of magnitude (Figure S27a,c, Supporting Information). PCD‐TPT + CsPbBr_3_/Cs_4_PbBr_6_ QDs photoFETs perform even better in this regard, with I_on_/I_off_ and mobility values remaining similar to the pristine PCD‐TPT transistors (Figure S27b,d, Supporting Information). These results provide evidence that this strategy is highly effective at both increasing the photosensitivity and maintaining good transistor operation even under illumination, paving the way for multifunctional optoelectronic devices.

## 4 Conclusion

In summary, this study has demonstrated a discrete phase transformation process in Cs_4_PbBr_6_ NCs, leading to creation of tunable hybrid CsPbBr_3_/Cs_4_PbBr_6_ QDs with adjustable luminescence across the blue, cyan, and green spectral regions achieved through the strategic kinetic trapping of the metastable phases. Furthermore, the research uncovered the unique chemical properties of the hybrid structures, including reversible interactions, iodide‐ion exchanges, and the role of CsBr leaching and dissolution‐recrystallization mechanisms. Furthermore, the photoFETs containing CsPbBr_3_/Cs_4_PbBr_6_ QDs blended with *n‐*type semiconducting polymers, exhibited increased performances in the dark and under illumination compared to phase‐pure CsPbBr_3_ NCs blends. The blended devices using the hybrid QDs could better harness the photogating effect of CsPbBr_3_ owing to its separation from the semiconducting polymer by the insulating Cs_4_PbBr_6_. Overall, our research highlighted the intricate interparticle interaction of colloidal Cs_4_PbBr_6_ NCs in regulating phase transformation for creating adjustable emissions in hybrid structures, and offers promising potential for the future development of photofield‐effect transistors.

## Conflict of Interest

The authors declare no conflict of interest.

## Author Contributions

U.T. conceived the idea and supervised the research. X.H. performed synthesis, optical, and morphological characterizations of the perovskites, M.P., and Y.W., assisted TEM characterizations, and analyzed the data. S.W., L.H., J.Z., and T.L. performed the photoFET fabrication and characterizations. S.W. and A.M. performed the CV characterizations. U.T., A.K.B., and T.L. wrote the paper. All authors discussed the contents and contributed to the manuscript.

## Supporting information

Supporting Information

## Data Availability

The data that support the findings of this study are available in the supplementary material of this article.
