# Supplementary material for: Tunable Emissive CsPbBr3/Cs4PbBr6 Quantum Dots Engineered by Discrete Phase Transformation for Enhanced Photogating in Field‐Effect Phototransistors
Source: Adv Sci (Weinh). 2024 Jun 21;11(32):2401973. doi: 10.1002/advs.202401973 (PMC11348058; doi:10.1002/advs.202401973)
Supplement: Supplementary file 1 — Supporting Information [file ADVS-11-2401973-s001.docx]

**Supporting Information**

| Serial order | Description | Page no. |
| --- | --- | --- |
| S1 | Experimental Section & Analytical Procedures | 2-3 |
|  | Table S1 and Table S2 | 5 |
| **Supporting Figures** | | |
| Fig. S1 | TEM images of pristine Cs_4_PbBr_6_ NCs | 6 |
| Fig. S2 | UV-vis spectra of pristine Cs_4_PbBr_6_ NCs | 6 |
| Fig. S3 | Comparison of UV-vis spectra as a function of dilution | 7 |
| Fig. S4 | XRD study of cyan emitting Cs_4_PbBr_6_/CsPbBr_3_ NCs | 7 |
| Fig. S5 | Schematic representation of phase transformation reaction | 8 |
| Fig. S6  Fig. S7  Fig. S8  Fig. S9  Fig. S10 | Photographs of the phase transformation reaction  Electroluminescence spectrum of the WLED device fabricated  Iodide-exchange reaction on cyan emitting hybrid NCs  Iodide-exchange reaction on green emitting hybrid NCs  Phase transformation kinetics in cyan emitting hybrid NCs | 8  9  9  10  11 |
| Fig. S11 | Reversible phase changes in cyan emitting hybrid NCs | 12 |
| Fig. S12 | PL spectra: influence of dilution in Cs_4_PbBr_6_ NCs (hexane) | 13 |
| Fig. S13 | PL spectra: influence of dilution in Cs_4_PbBr_6_ NCs (ODE) | 13 |
| Fig. S14  Fig. S15 | PL spectra: influence of dilution in Cs_4_PbBr_6_ NCs (Toluene)  XRD study of green emitting mixed phases | 14  14 |
| Fig. S16 | TEM and XRD analysis of CsPbBr_3_ NCs | 15 |
| Fig. S17 | UV-vis spectra: influence of dilution in Cs_4_PbBr_6_ NCs | 16 |
| Fig. S18 | UV-vis spectra: concentrated and diluted in Cs_4_PbBr_6_ NCs | 17 |
| Fig. S19  Fig. S20 | TEM images of dilution Cs_4_PbBr_6_ NCs in hexane  TEM images of dilution Cs_4_PbBr_6_ NCs in toluene | 18  19 |
| Fig. S21 | TEM images of the synthesized Cs_4_PbBr_6_ NCs in ODE | 20 |
| Fig. S22 | TEM images of dilution Cs_4_PbBr_6_ NCs in hexane | 20 |
| Fig. S23 | DLS spectra of dilution Cs_4_PbBr_6_ NCs | 21 |
| Fig. S24 | Optoelectrical studies of transistors of CsPbBr_3_ and hybrid NCs. | 21 |
| Fig. S25 | Cyclic Voltammetry curves of CsPbBr_3_ and hybrid NCs | 22 |
| Fig. S26 | PhotoFET studies: CsPbBr_3_ and hybrid NCs polymer composites | 22 |
| Fig. S27 | PhotoFET studies: CsPbBr_3_ and hybrid NCs polymer composites | 23 |
|  |  |  |
|  |  |  |
|  |  |  |
|  |  |  |
|  |  |  |

**S1-Experimental Procedures**

**Materials.** PbBr_2_ (lead (II) bromide 99.999 %, Alfa Aesar (China) Chemical Co., Ltd.), Cs_2_CO_3_ (99.99 %, Shanghai Aladdin Biochemical Technology Co., Ltd., China), 1-octadecene (ODE, 90 %，Shanghai Aladdin Biochemical Technology Co., Ltd., China), oleic acid (analytical grade, Shanghai Aladdin Biochemical Technology Co., Ltd., China), oleylamine (95 %, Shanghai Aladdin Biochemical Technology Co., Ltd., China), n-hexane and toluene (97%, Shanghai Macklin Biochemical Co., Ltd, China), methyl acrylate (MA, 99.5 %, Shanghai Macklin Biochemical Co., Ltd, China). All the chemical reagents were used as received without further purification.

Preparation of Cs*-*oleate*.* Cs_2_CO_3_ (0.814 g), 1-octadecene (40 mL) and oleic acid (2.5 ml,1.5 mmol) were added into a 100 mL three-necked flask dried for 1 hour at 100 ℃, then raised the temperature to 150 ℃ under nitrogen until Cs_2_CO_3_ and oleic acid were reacted completely.

Synthesis of Cs_4_PbBr_6_ NCs. PbBr_2_ (0.188 mmol, about 0.069 g) and 1-octadecene (5 mL) were mixed into a 25 mL three-necked flask and reacted 1 hour at 100 ℃ and stirred at 1000 in a vacuum environment. Dried Oleyl amine (1.1 mL,1.5 mmol) and dried OA (1 ml,1.5 mmol) were injected at the same time at 100 ℃ under N_2_. When the solution changed from cloudy white to clear yellow, the temperature was raised to 120 ℃ and Cs-oleate solution (0.4 mL, 0.05 mmol, 0.125 M in ODE, pre-heated to 70 ℃) was quickly injected. Then the reaction was cooled by the ice-water bath at the same time. The solution was continued to stir at 400 rpm. In our synthetic reaction, we have used a slight excess of oleylamine. It is expected that the oleylamine plays a key role in triggering the formation of Cs4PbBr6 NCs, however the oleylamine alone could produce only polydisperse NCs. The presence of amine-oleic acid ligand combination will greatly improve the size uniformity. ^[1.2]^ From TEM, we calculated the mean diameter of the Cs_4_PbBr_6_ NCs ( ∼ 11 nm). Then we centrifuged 1 mL of samples to get the weight of Cs_4_PbBr_6_ NCs. Because volume is equal to (4/3) *Π*r^3^ and the density of Cs_4_PbBr_6_ NCs is 4.19 g/cm^3^. So, we can calculate the number of Cs_4_PbBr_6_ NCs in 1 mL which is 1.43*10^22^/mL.

Effect of Dilution on transformation temperature of Cs_4_PbBr_6_ NCs. The Cs_4_PbBr_6_ NCs were diluted with ODE (the volume ratio for both two materials are equal to 1:1) and then heated at 110 °C until the blue emission occurred. Meanwhile, the Cs_4_PbBr_6_ NCs without dilution are also heated at 110 °C, which doesn’t have emission during the heating process. To test whether the temperature will influence the result, the O-Cs_4_PbBr_6_ NCs were heated at 140 °C, then the green emission appeared in the system.

Synthesis of tunable emissions in Cs_4_PbBr_6_ NCs. Specifically, different amounts of Cs_4_PbBr_6_ (gradient growth from 20 μL to 200 μL, O_d_ to O_100d_) were mixed with 2 mL hexane (99 %). Then dilution samples were placed on a heating plate and heated at 70 °C. Observing the color change of the solution under UV light. All experimental groups had no fluorescence at first. With the increasing heating time, fluorescence gradually began to appear. When any of the three colors of green, cyan, and blue appeared in the experimental group, removing the emission sample from the heating plate to stop heating. Upon the precipitation, and redispersion, the colloidal green and cyan emitting QDs are stable for several days to weeks, whereas the blue emitting QDs turns to non-emissive. Over all the green emissive QDs is highly stable for months in solution as well as in solid state.

Spontaneous phase transformation in Cs_4_PbBr_6_ NCs. The Cs_4_PbBr_6_ NCs were diluted in different solvents (toluene, ODE, and hexane) at room temperature. During the dilution process, we should notice the UV/vis peak, PL spectra peak, and TEM image changing of these samples.

Transmission electron microscope (TEM) images: The morphology of the NCs was obtained by a FEI TECNAI G2 F20 TEM operating at 200 kV.

X-ray diffraction (XRD) patterns: XRD patterns of NCs were carried out using a desktop diffractometer (D2 PHASER, Bruker, Germany) with a Cu K_α_ source.

Optical properties characterization: UV/vis absorption spectra were measured by using a Shimadzu UV-1900 spectrophotometer in a range of 200-800 nm. The photoluminescence (PL) spectra were performed on a fluorescence spectrophotometer (FLUOROMAX-4). The Photoluminescence quantum yield (PLQY) and lifetime measurement were collected by using the fluorescence spectrophotometer (FLS-1000).

**Device fabrication:** Interdigitated bottom-contact bottom-gate configuration transistors consisted of n++-Si substrates with 200 nm of thermally grown SiO_2_ as the gate dielectric and patterned pairs of gold electrodes with interdigitated geometry as the source and drain (W = 10 mm, L = 20 µm, Ti adhesion layer). All solutions, samples and devices were prepared and measured in a nitrogen filled glovebox to avoid oxidative doping and degradation of the materials and ensure reproducibility of the experiments. All wafers were treated with octadecyltrichlorosilane (OTS) before use (see below). Samples with organic semiconductors in the channel were fabricated by spin-coating of the semiconductor or semiconductor/perovskite solution at a total concentration of 5 mg/mL, at 3000 RPM. The semiconductors were purchased from Derthon (P(NDI2OD-T2)) and 1-Material (PCDTPT).

Pure perovskite samples were fabricated by deposition via drop-casting of 100 µL of a 10 mg/mL perovskite solution. All the solutions were prepared using chloroform as solvent.

OTS treatment consisted of an overnight immersion of the UV-ozone cleaned substrate into a 1 millimolar solution of OTS in toluene starting with a 1-hour heating of the solution at 60°C. The overnight immersion was followed by rinsing with clean toluene and annealing of the clean substrates for 30 minutes at 60°C to remove all traces of solvent. The immersion, rinsing, and annealing steps were performed in a nitrogen atmosphere.

**Device characterization**: Transistor properties were evaluated either under positive or negative gate bias to explore the majority charge carrier type and device performance (positive bias for P(NDI2OD-T2) containing devices and negative bias for PCDTPT containing devices. Transfer curves were obtained by measuring the IDS as a function of the gate bias from -60 V to +60 V (P(NDI2OD-T2)) and from +60 V to -60 V (PCDTPT) with one point measured every 2 V, using a Keithley 2636B sourcemeter. All electrical characterization was performed in a nitrogen environment. Mobility values were obtained using the standard field-effect transistor equations in the saturation regime, i.e., at V_DS_ = ±60 V from the slope of plots of (IDS)^1/2^ versus V_GS_.

**Cyclic voltammetry measurements:**

Cyclic voltammetry measurements were performed with SP-300 (Biologic) potentiostat in a standard three electrode cell setup using acetonitrile and 0.1 M tetrabutylammoniumhexaflorophosphate (TBAPF6) as electrolyte. Nanocrystals and polymers were deposited on FTO coated glass, which served as a working electrode. Pt wire was used as a counter electrode and another Pt wire was used as reference electrode. Potential was adjusted versus ferrocenium/ferrocene redox couple. The HOMO and LUMO of the nanocrystals and the polymers were measured by Cyclic Voltammetry, as shown in **Fig. S24**. According to the formulas ^[31]^:

(2)

(3)

The HOMO of CsPbBr_3_ is -5.93 eV and LUMO of CsPbBr_3_ is -3.58 eV, and the HOMO Cs_4_PbBr_6_/CsPbBr_3_ hybrid is -5.96 eV and LUMO of Cs_4_PbBr_6_/CsPbBr_3_ hybrid is -3.58 eV. And we can get the HOMO and LUMO values of P(NDI2OD-T2), PCD-TPT, as shown in Table S1.

|  | HOMO (eV) | LUMO (eV) |
| --- | --- | --- |
| P(NDI2OD-T2) | -5.75 | -4.14 |
| PCD-TPT | -5.38 | -4.08 |

**Table S1:** HOMO and LUMO values of P(NDI2OD-T2), PCD-TPT.

|  | µ_dark_ / cm²/Vs | µ_illumination_/ cm²/Vs | I_on_/I_off,dark_ | I_on_/I_off,illumination_ | P |
| --- | --- | --- | --- | --- | --- |
| P(NDI2OD-T2) | 1.19 × 10^-2^ | 1.15 × 10^-2^ | 3 × 10^7^ | 2 × 10^6^ | 2.81× 10^1^ |
|  |  |  |  |  |  |
| P(NDI2OD-T2)  + CsPbBr_3_ NCs | 6.76 × 10^-5^ | 2.26 × 10^-5^ | 2 × 10^5^ | 1 × 10^4^ | 6.46 × 10^3^ |
| P(NDI2OD-T2)  + CsPbBr_3_/Cs_4_PbBr_6_ NCs | 2.17 × 10^-4^ | 4.94 × 10^-4^ | 3 × 10^5^ | 1 × 10^4^ | 1.85 × 10^5^ |
|  |  |  |  |  |  |
| PCD-TPT | 2.35 × 10^-3^ | 2.37 × 10^-3^ | 2 × 10^3^ | 2 × 10^3^ | 1.61 × 10^2^ |
|  |  |  |  |  |  |
| PCD-TPT  + CsPbBr_3_ NCs | 1.54 × 10^-3^ | 1.29 × 10^-3^ | 5 × 10^6^ | 3 × 10^5^ | 1.18 × 10^3^ |
|  |  |  |  |  |  |
| PCD-TPT  + Cs_4_PbBr_6_/CsPbBr_3_ NCs | 5.11 × 10^-3^ | 5.01 × 10^-3^ | 3 × 10^7^ | 7 × 10^8^ | 2.50 × 10^3^ |

**Table S2:** Field-effect phototransistor characterization of the devices based on organic semiconductors and blends containing organic semiconductors and NCs (values extracted based on **Fig. S25**).

**Supplementary Figures**

**The properties of pure Cs_4_PbBr_6_ NCs.**


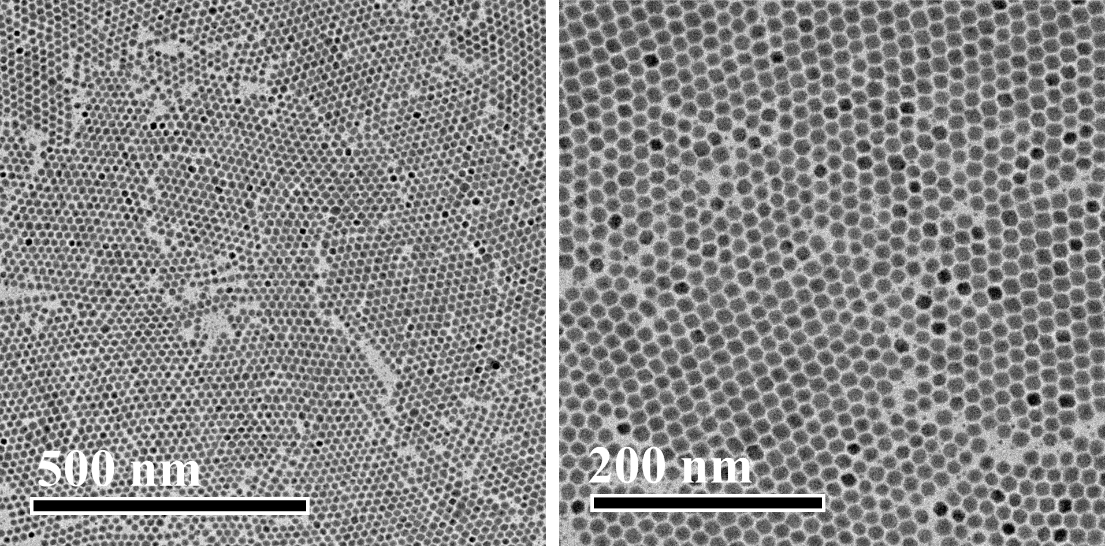


**Fig. S1.** TEM images of as-synthesized monodisperse non-emissive Cs_4_PbBr_6_ NCs at different magnifications.

**
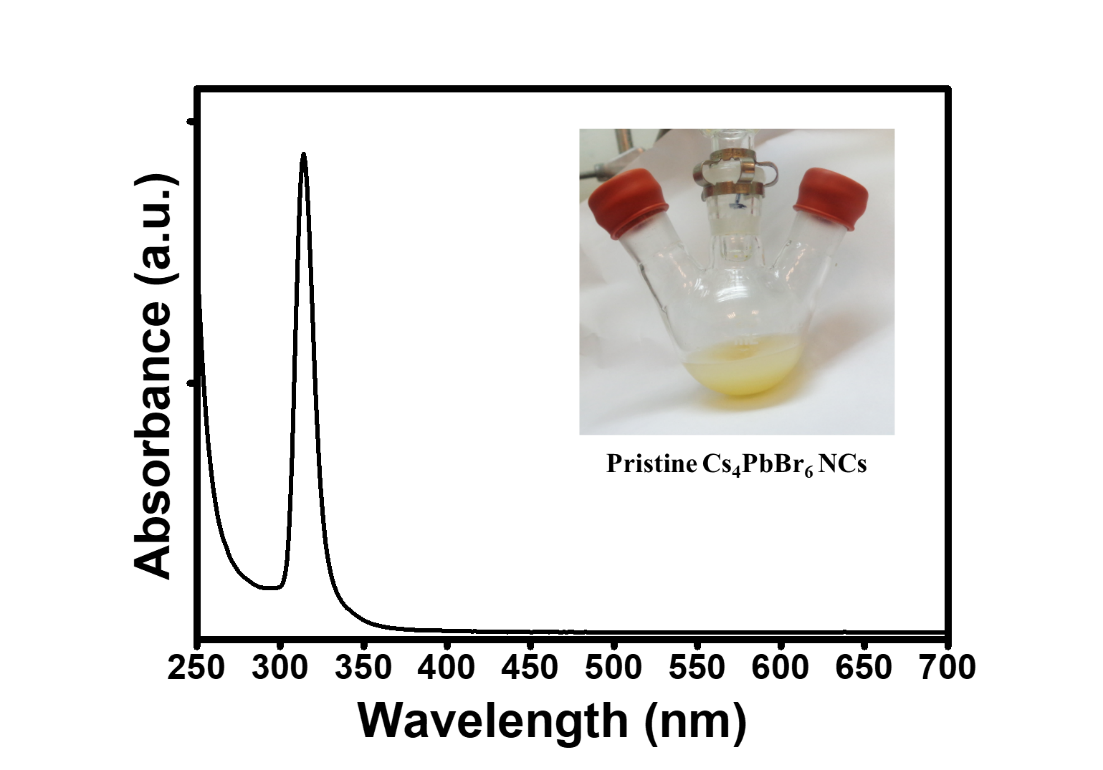
**

**Fig. S2.** The UV/vis absorption spectrum of as-synthesized Cs_4_PbBr_6_ NCs. Inset show the photographs of the as-synthesized Cs_4_PbBr_6_ NCs under day light.

**Fig. S3.** The normalized UV/vis absorption spectra of the Au NCs (a) and Cs_4_PbBr_6_ NCs (b) at different dilutions, two times (O_2d_), five times (O_5d_) and a hundred times (O_100d_).

**
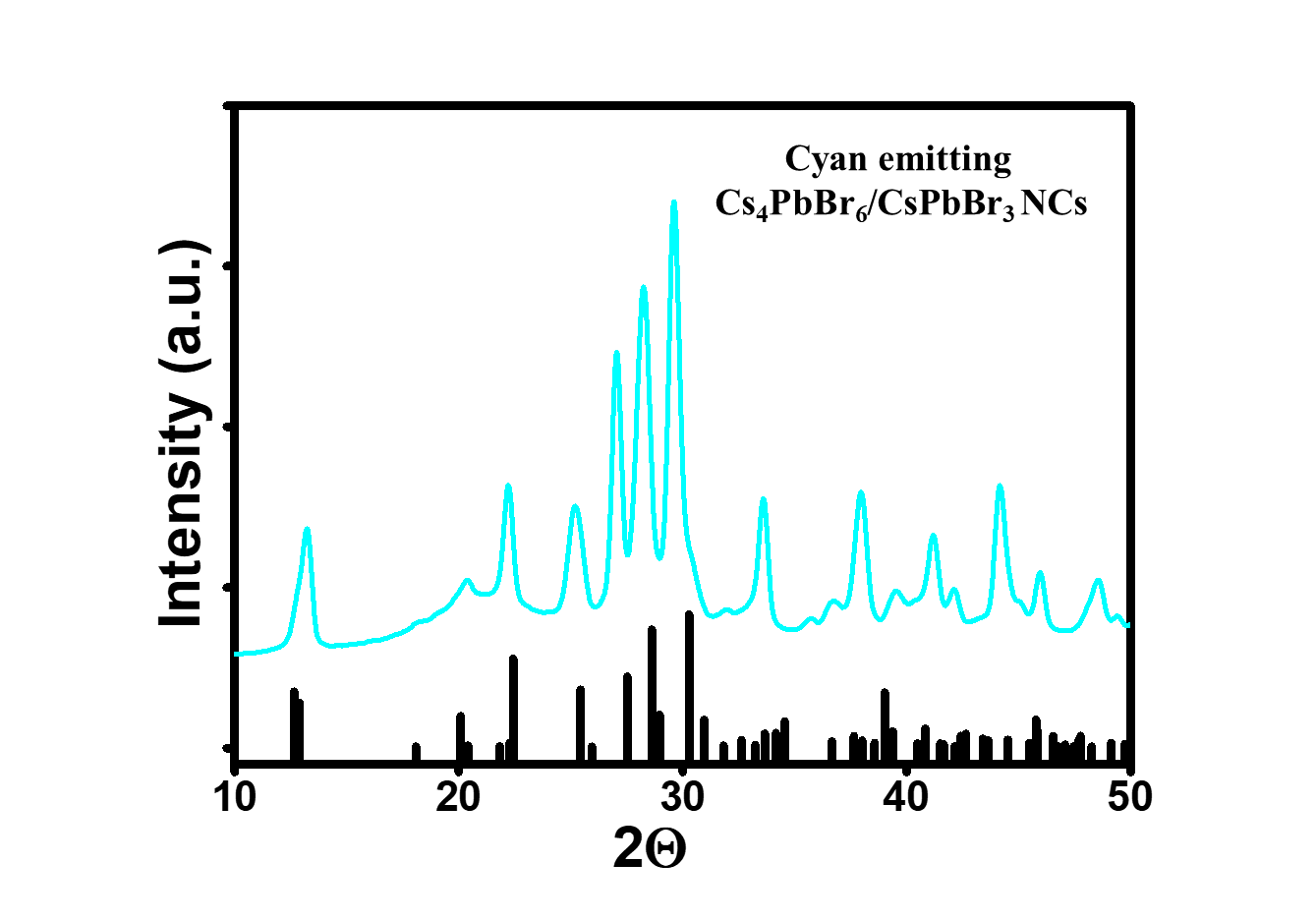
**

**Fig. S4.** X-ray diffraction pattern of cyan emitting CsPbBr_3_/Cs_4_PbBr_6_ QDs.


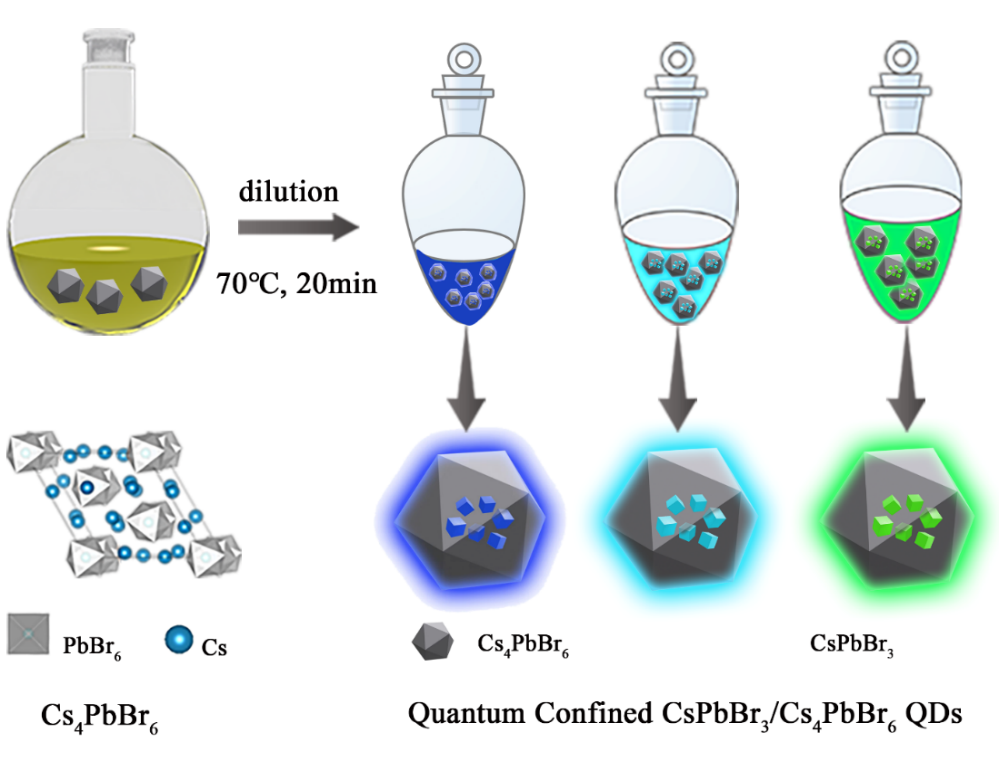


**Fig. S5.** Schematic illustration of fabrication of tunable emission in CsPbBr_3_/Cs_4_PbBr_6_ QDs. When the concentration of Cs_4_PbBr_6_ NCs is high, the sample has a blue emission, and as the concentration is decreasing, the emission color will turn to cyan, and finally turn to green.


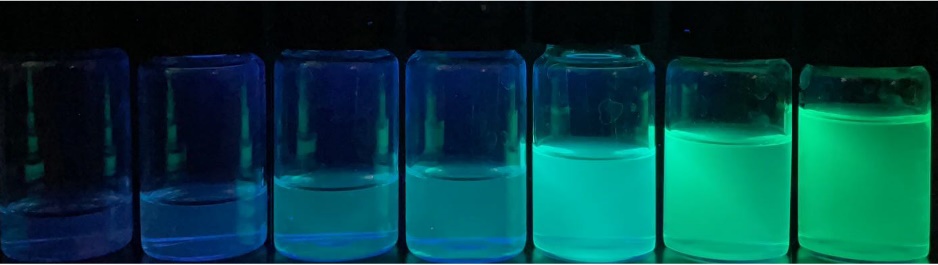


**Fig. S6.** Photographs of tunable emission from CsPbBr_3_/Cs_4_PbBr_6_ QDs through dilution-controlled phase transformation reaction in Cs_4_PbBr_6_ NCs.

**Fig. S7.** Electroluminescence spectrum of the WLED device fabricated using green emitting CsPbBr_3_/Cs_4_PbBr_6_ QDs under different driving currents.

**
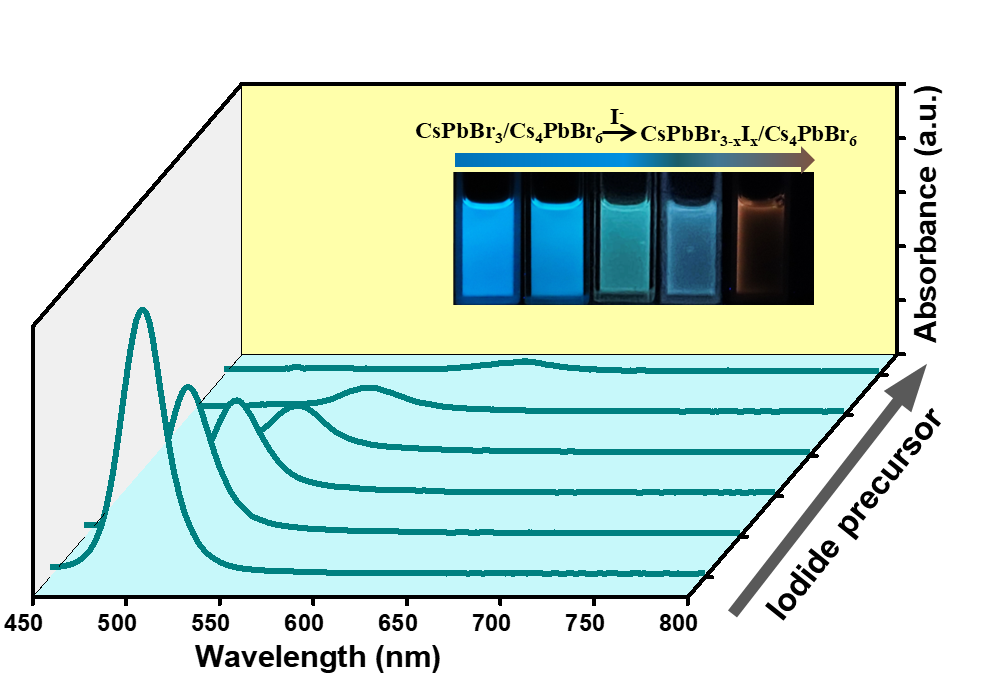
**

**Fig. S8.** A systematic change in PL wavelength up on the Br^-^ to I^-^ exchange in cyan emitting CsPbBr_3_/Cs_4_PbBr_6_ QDs. Inset show the photographs of the respective solutions under UV light.

**Fig. S9.** A systematic change in PL wavelength up on the Br^-^ to I^-^ exchange in green emitting CsPbBr_3_/Cs_4_PbBr_6_ QDs. Inset show the photographs of the respective solutions under UV light.


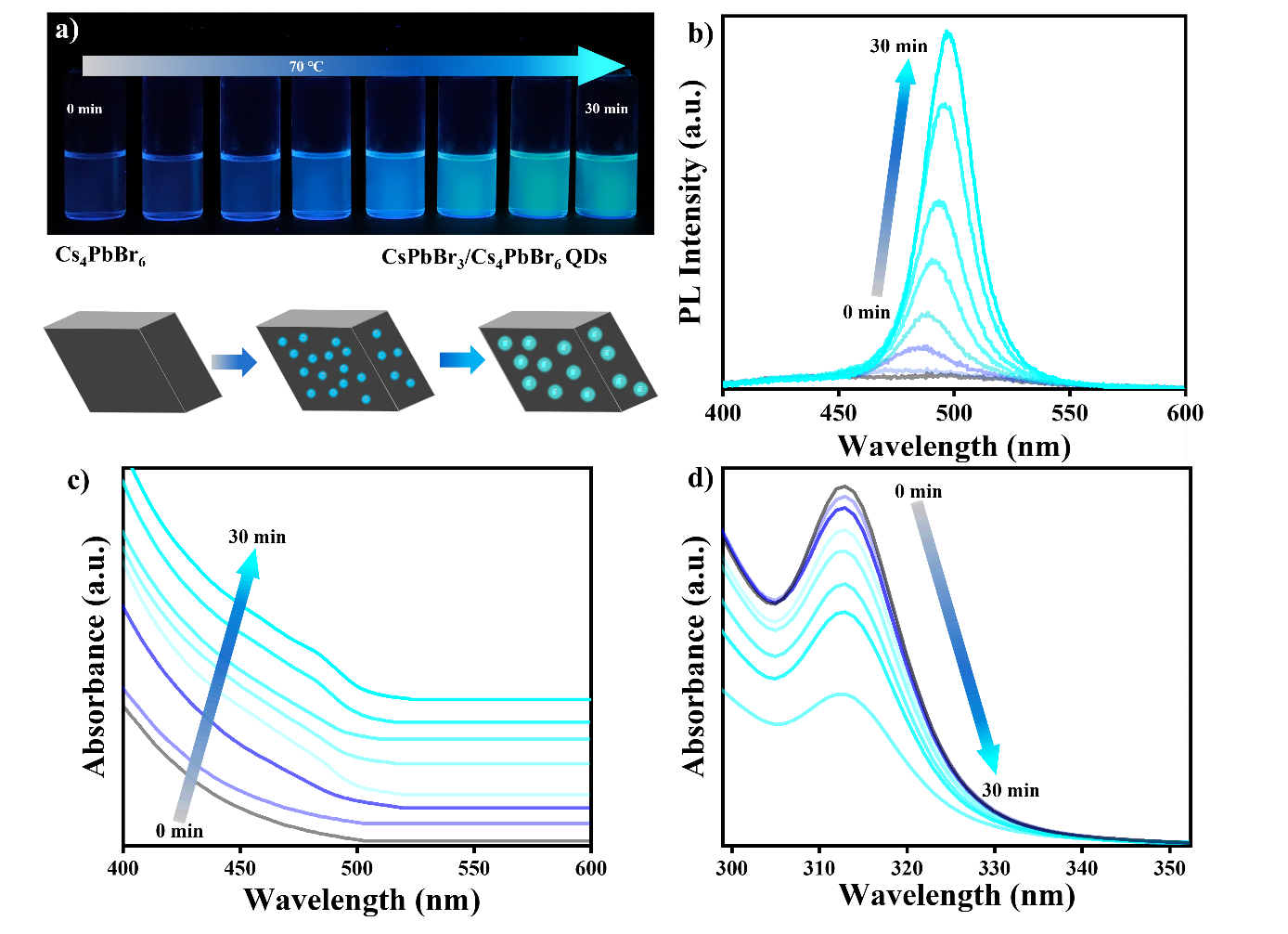


**Fig. S10:** Systematic study of phase transformation of non-emissive Cs_4_PbBr_6_ NCs to cyan-emitting CsPbBr_3_/Cs_4_PbBr_6_ QDs. (a) Photographs of the Cs_4_PbBr_6_ NCs in hexane (O_5d_) collected over 30 min while the solution was heated at 70 °C, respective PL emission (b) and absorption spectra (c and d).


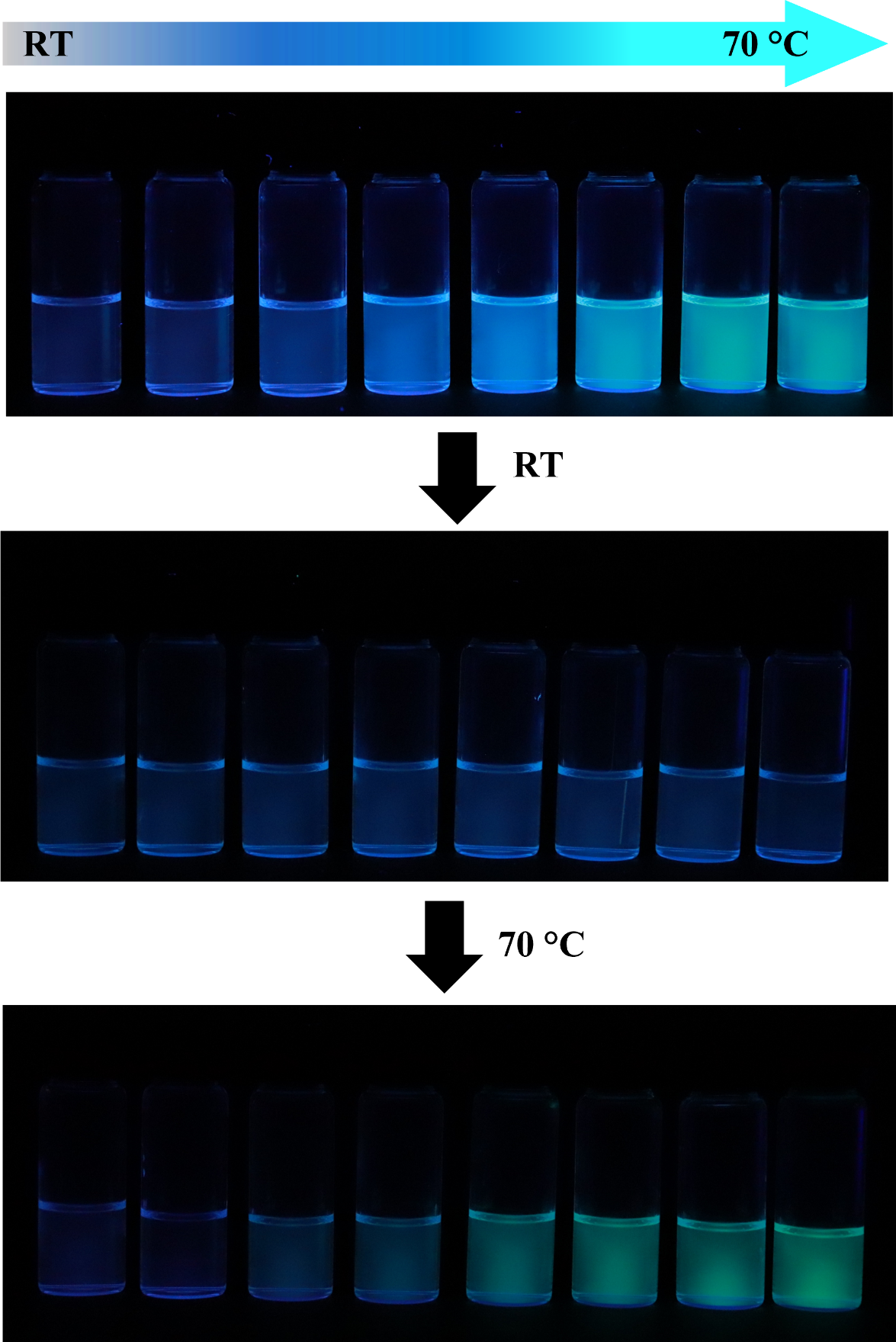


**Fig. S11:** Illustration of reversible phase transformation of non-emissive Cs_4_PbBr_6_ NCs to cyan-emitting CsPbBr_3_/Cs_4_PbBr_6_ NCs by their respective photographs collected under UV light.

**Fig. S12.** A systematic change to the PL spectra upon diluting the non-emissive Cs_4_PbBr_6_ NCs with hexane.

**Fig. S13.** A systematic change to the PL spectra upon diluting the non-emissive Cs_4_PbBr_6_ NCs with octadecane.

**Fig. S14.** A systematic change to the PL spectra upon diluting the non-emissive Cs_4_PbBr_6_ NCs with toluene.


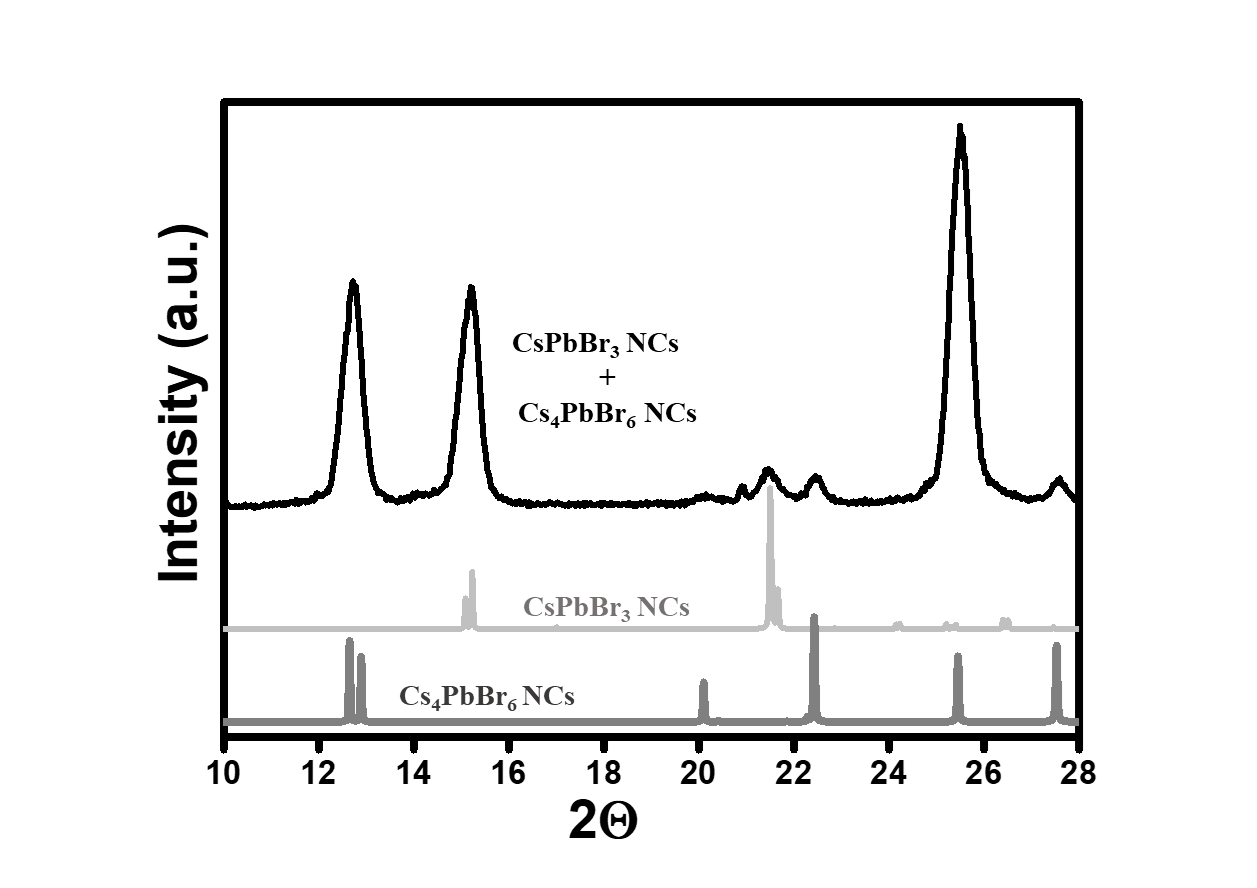


**Fig. S15.** X-ray diffraction pattern of green emitting mixed phases **(**CsPbBr_3_ NCs and Cs_4_PbBr_6_ NCs) obtained through the spontaneous dilution.

**
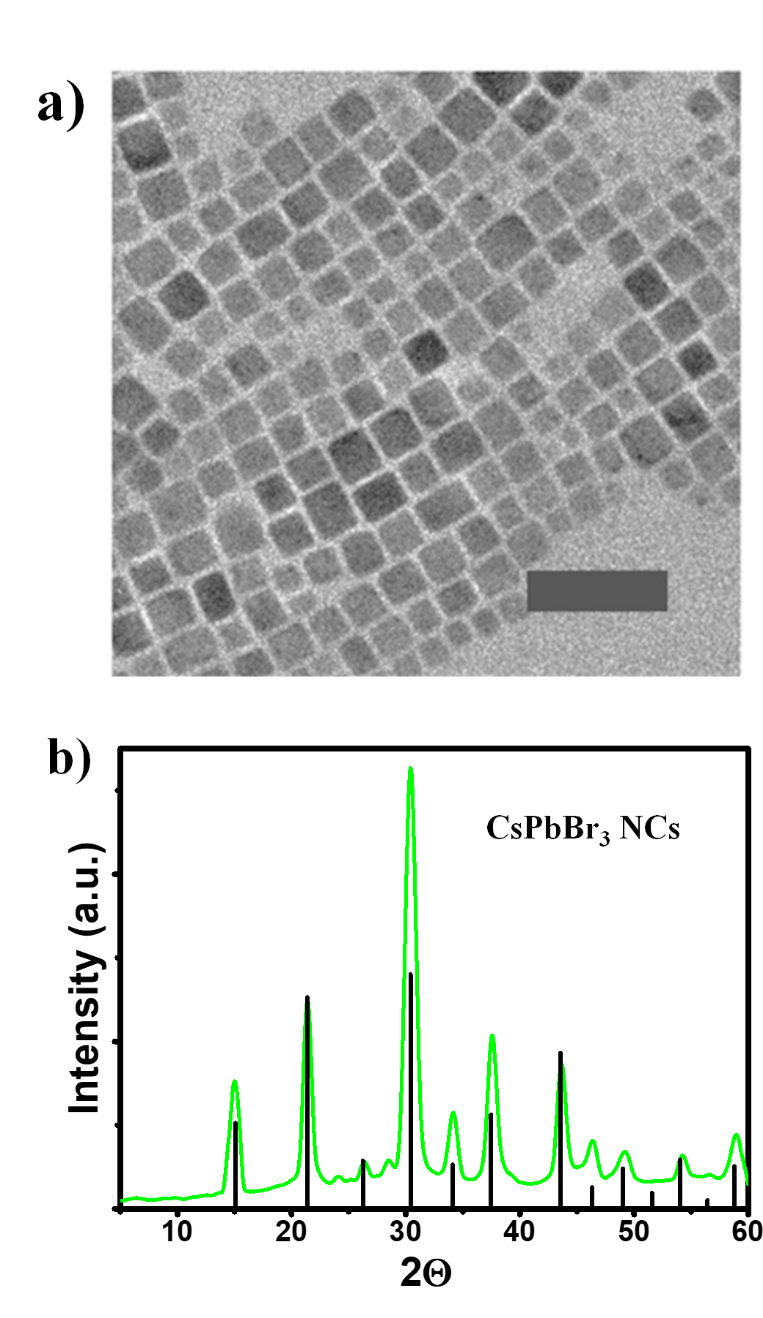
**

**Fig. S16.** TEM (a) and XRD (b) of CsPbBr_3_ NCs prepared through the spontaneous phase transformation of Cs_4_PbBr_6_ NCs to CsPbBr_3_ NCs at higher toluene dilutions at room temperature. Scale bar: 50 nm

**Fig. S17.** The systematic change in UV/vis absorption spectra upon dilution of Cs_4_PbBr_6_ NCs by (a) hexane, (b) ODE, and (c) toluene.


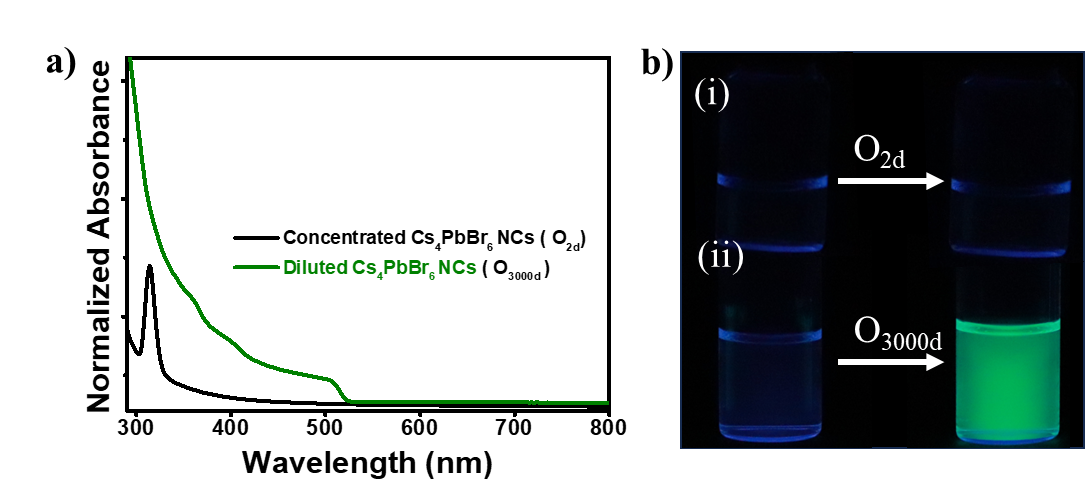


**Figure S18:** (a) The UV/vis absorption spectra show the optical properties of Cs_4_PbBr_6_ NCs at different dilutions. (b) The photographs depict the physical appearance of Cs_4_PbBr_6_ NCs at concentrated and higher dilutions in hexane under UV light. At concentrated Cs_4_PbBr_6_ NCs, no phase transformation is observed, whereas a spontaneous phase transformation from 0D to 3D occurs at higher dilutions.


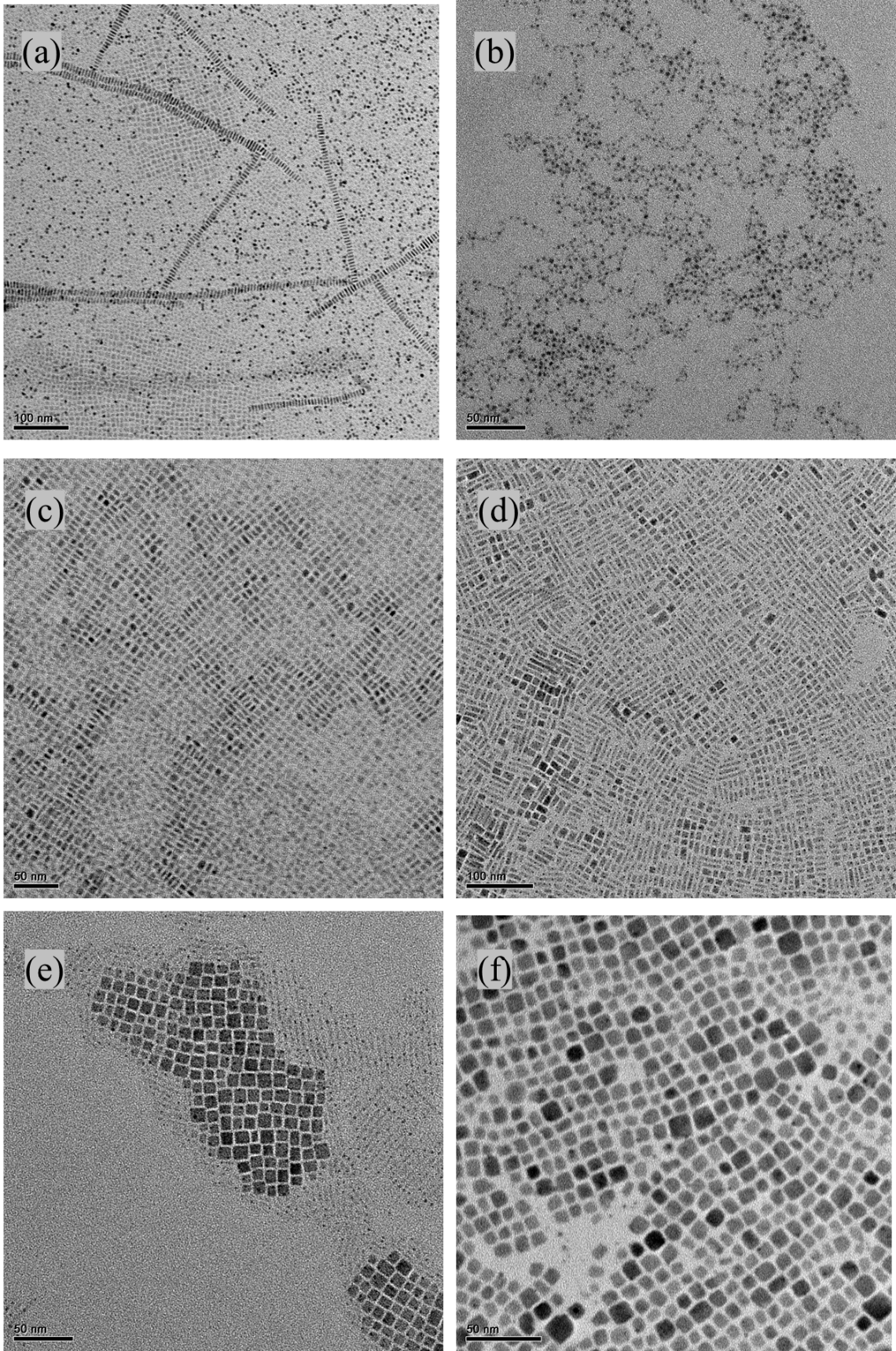


**Fig. S19.** TEM images show the morphological changes (from CsPbBr_3_ nanoplatelets to nanocubes) that occurred during the dilution of the Cs_4_PbBr_6_ NCs with hexane.


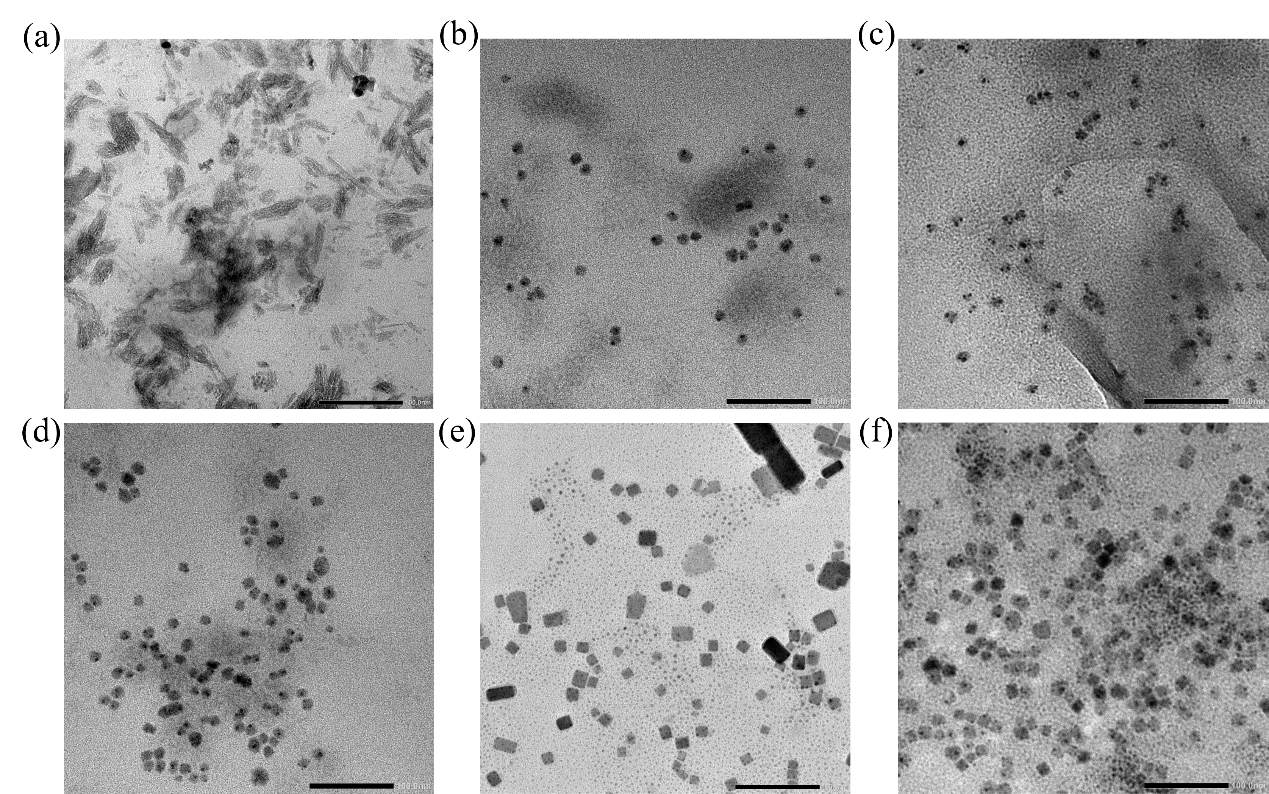


**Fig. S20.** TEM images show the morphological changes that occurred during the dilution of the Cs_4_PbBr_6_ NCs with toluene. (a), (b) demonstrate the initial stages of dissolution in the diluted Cs_4_PbBr_6_ NCs process. (c) and (d) illustrate the hybrid process that occurred during the dilution. (e) and (f) showcase the recrystallization process that took place during the dilution.


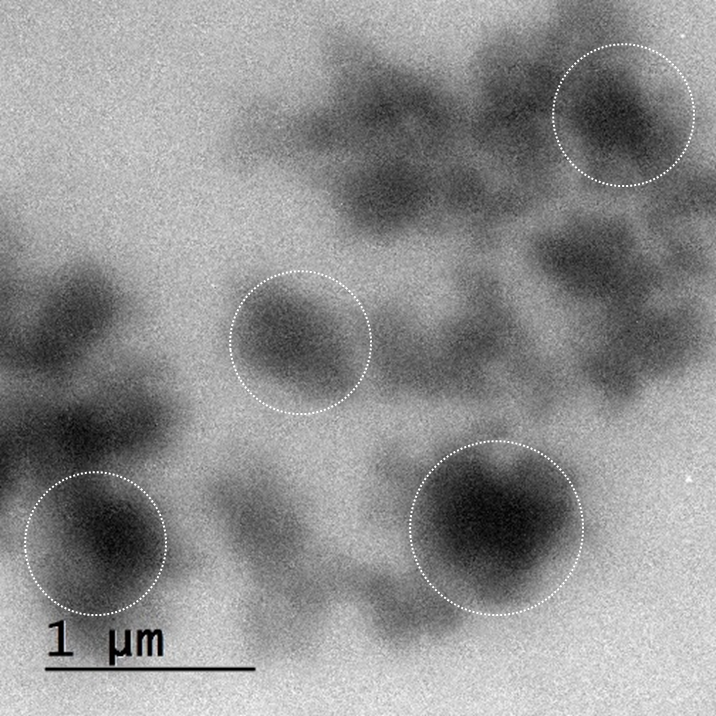


**Fig. S21.** TEM images of Cs_4_PbBr_6_ NCs synthesized in octadecane. Despite the poor image quality due to the oily nature of octadecane, the images reveal the intact state of the NCs within the cloudy solutions, where they exist as aggregates with micron-sized dimensions. These aggregates exhibit a broad absorption spectrum.


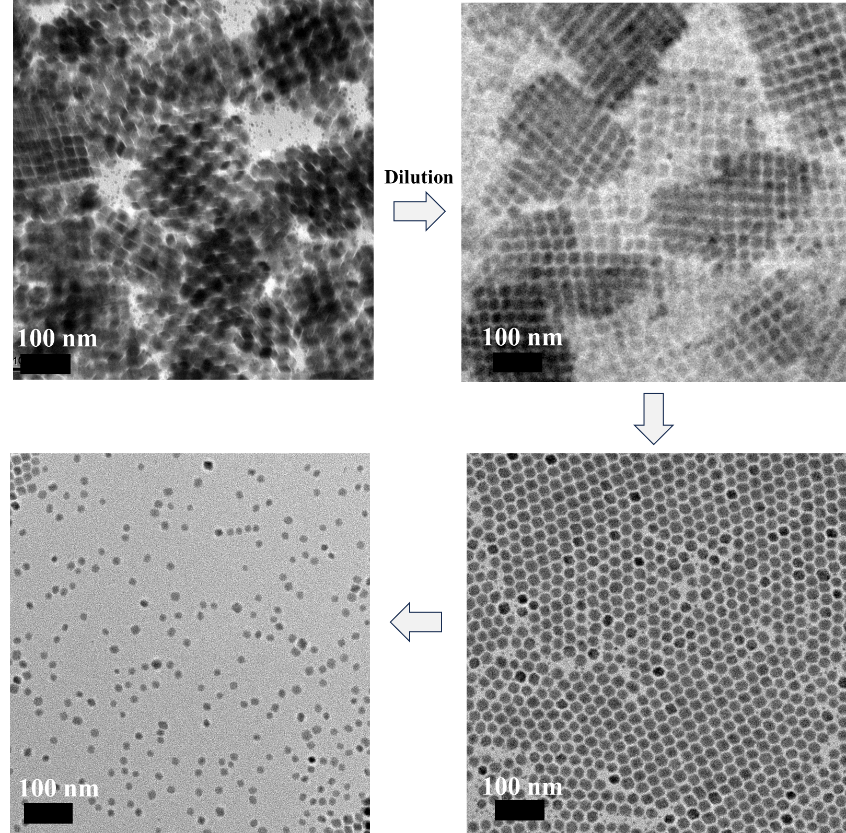


**Fig. S22.** TEM images of the as-synthesized Cs_4_PbBr_6_ NCs were subjected to different dilutions.


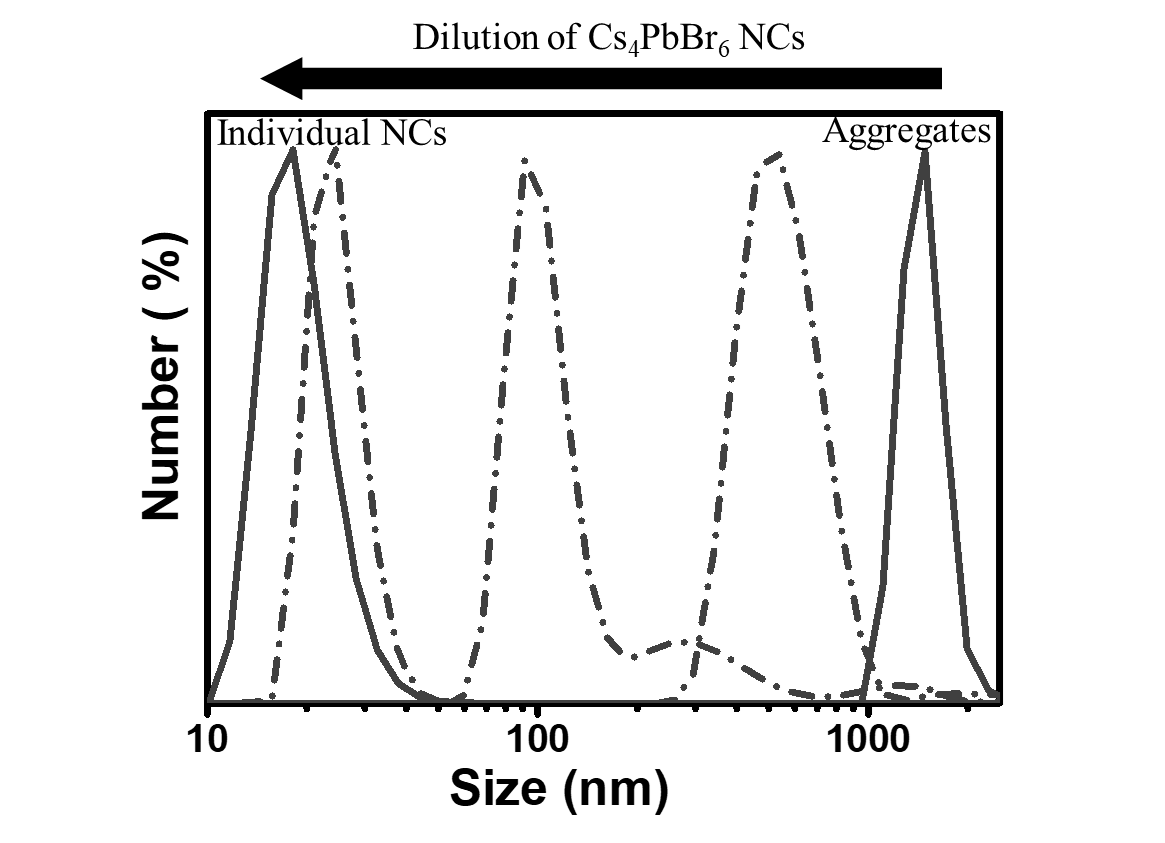


**Fig. S23.** DLS spectra of Cs_4_PbBr_6_ NCs at different dilutions in hexane show the transformation of aggregates to individual NCs.

**Fig. S24**: Optoelectrical characterization of transistors with pure CsPbBr_3_ NCs and CsPbBr_3_/Cs_4_PbBr_6_ QDs films in the channel. All curves for devices with L = 20 µm and W = 10 mm at V_DS_ = -60 V. **(a**) Transfer curves of photoFETs based on CsPbBr_3_ NCs, in the dark and under illumination at 420 nm. (b) Transfer curves of photoFETs based on CsPbBr_3_/Cs_4_PbBr_6_ QDs, in the dark and under illumination at 420 nm.

**Fig. S25**: Cyclic Voltammetry curves of (a) CsPbBr_3_ NCs and CsPbBr_3_/Cs_4_PbBr_6_ QDs films, (b) P(NDI2OD-T2) films and (c) PCDTPT films.

**Fig. S26**: Electrical and optoelectrical characterization of the photoFETs. All curves for devices with L = 20 µm and W = 10 mm at V_DS_ = -60 V. a) Transfer curves of photoFETs based on the blends P(NDI2OD-T2) + CsPbBr_3_ NCs and P(NDI2OD-T2) + CsPbBr_3_/Cs_4_PbBr_6_ QDs, in the dark and under illumination at 420 nm. b) Transfer curves of photoFETs based on the blends PCD-TPT + CsPbBr_3_ NCs and PCD-TPT + CsPbBr_3_/Cs_4_PbBr_6_ QDs in the dark and under illumination at 420 nm.

**Fig. S27**: Electrical and optoelectrical characterization of photoFETs based on pure organic semiconductors. All curves for devices with L = 20 µm and W = 10 mm at V_DS_ = -60 V. Transfer curves of FETs in the dark and under illumination at 420 nm. a) P(NDI2OD-T2), linear scale. b) PCD-TPT, linear scale. c) P(NDI2OD-T2), logarithmic scale. d) PCD-TPT, logarithmic scale.

**References:**

1. Z. Liu, Y. Bekenstein, X. Ye, S. C. Nguyen, J. Swabeck, D. Zhang, S-T. Lee, P. Yang, W. Ma, A.P. Alivisatos, *J. Am. Chem. Soc.* **2017**, 139, 5309-5312.
2. T. Udayabhaskararao, L. Houben, H. Cohen, M. Menahem, I. Pinkas, L. Avram, T. Wolf, A. Teitelboim, M. Leskes, O. Yaffe, D. Oron, *Chemistry of Materials*, **2018**, 30*,* 84-93.
